# Four New Sesquiterpenoids from Cultures of the Fungus *Phellinidium sulphurascens*

**DOI:** 10.1007/s13659-014-0047-x

**Published:** 2014-12-10

**Authors:** Zhen-Zhu Zhao, He-Ping Chen, Tao Feng, Zheng-Hui Li, Ze-Jun Dong, Ji-Kai Liu

**Affiliations:** 1 State Key Laboratory of Phytochemistry and Plant Resources in West China, Kunming Institute of Botany, Chinese Academy of Sciences, Kunming, 650201 People’s Republic of China; 2 University of Chinese Academy of Sciences, Beijing, 100049 People’s Republic of China

**Keywords:** *Phellinidium sulphurascens*, Drimane, 7,10-Epoxy-2,6,10-trimethyldodeca-2,11-diene

## Abstract

Four new sesquiterpenoids, namely 12-hydroxy-3-oxodrimenol (**1**), 11-hydroxyacetoxydrim-7-en-3*β*-ol (**2**), 2,6-dimethyl-7,10-epoxy-10-hydroxymethyldodeca-2,11-dien-6-ol (**3**), and 7,10-epoxy-2,6,10-trimethyldodeca-2,11-diene-4,6-diol (**4**), along with fourteen known compounds, were isolated from the cultures of *Phellinidium sulphurascens.* The structures of compounds **1**–**4** were established on the basis of extensive spectroscopic analysis. All of them were evaluated for their cytotoxic activities.

**Electronic supplementary material:**

The online version of this article (doi:10.1007/s13659-014-0047-x) contains supplementary material, which is available to authorized users.

## Introduction

**Fig. 1:**
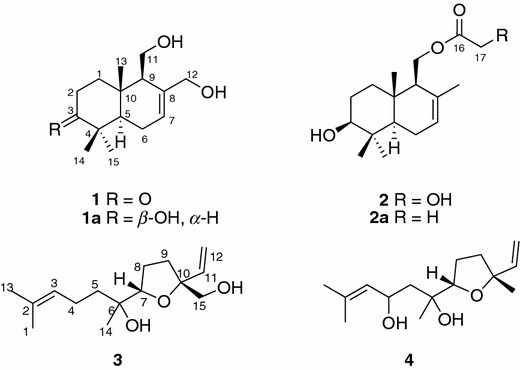
Structures of compounds **1**–**4**, **1a**, and **2a**

The wood-decaying fungus *Phellinidium sulphurascen* is a rare species belonging to the genus *Phellinidium* which is close-related to the genus *Phellinus* taxonomically. So far, only seven species of this genus were discovered in China, including *P. sulphurascens* [1]. In addition, the genus *Phellinus* has plenty of bioactive secondary metabolites involved in different types, such as sesquiterpenoids, steroids, pigments and polysaccharides [2–5]. Most of these metabolites showed significant vascular-relaxing [2], antiviral [3], estrogenic and anti-estrogenic [4], and antitumor activities [5]. However, the fungus *P. sulphurascens* has not been chemically investigated until now. As our continuous search for bioactive natural products from higher fungi, it is of importance to carry out the chemical investigation on cultures of *P. sulphurascens*, which resulted in the isolation of four new sesquiterpennoids (Fig. 1), namely 12-hydroxy-3-oxodrimenol (**1**), 11-hydroxyacetoxydrim-7-en-3*β*-ol (**2**), 2,6-dimethyl-7,10-epoxy-10-hydroxymethyldodeca-2,11-dien-6-ol (**3**), and 7,10-epoxy-2,6,10-trimethyldodeca-2,11-diene-4,6-diol (**4**), together with fourteen known compounds, from the EtOAc extract of the cultures of this fungus. All compounds were evaluated for their cytotoxicities against five human cancer cell lines.

## Results and Discussion

**Fig. 2:**
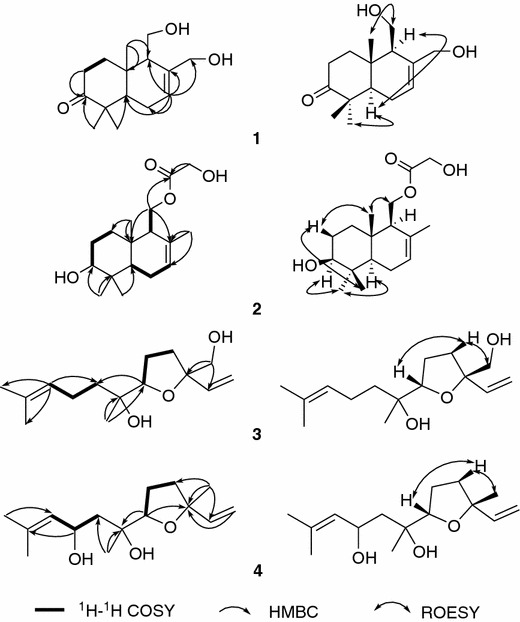
Key 2D NMR correlations of **1**–**4**

Compound **1**, obtained as amorphous powder, possessed a molecular formula C_15_H_24_O_3_, as deduced from HREIMS at *m/z* 252.1723 [M]^+^(calcd for C_15_H_24_O_3_, 252.1725). The IR spectrum showed absorption bands at 3440 cm^−1^ for hydroxy and 1701 cm^−1^ for carbonyl functional groups. The ^1^H NMR spectrum of compound **1** showed three methyl singlets (*δ*_H_ 1.02, 1.04 and 1.08), two *O*-bearing methylene signals (*δ*_H_ 3.70, ddd, *J* = 11.1, 5.0, 2.5 Hz; *δ*_H_ 3.90, ddd, *J* = 11.1, 6.9, 5.0 Hz and *δ*_H_ 3.96, dd, *J* = 12.2, 6.9 Hz; *δ*_H_ 4.27, dd, *J* = 12.2, 5.3 Hz), and one olefinic proton (*δ*_H_ 5.78, d, *J* = 5.1 Hz). The ^13^C NMR spectrum of **1** showed fifteen carbon resonances, which were ascribed to a trisubstituted double bond, three methyl, five methylenes (two oxygenated), two *sp*^3^ methines, two *sp*^3^ quaternary carbons and one carboxyl (*δ*_C_ 215.1) (Table 1). The chemical shift values of 1D NMR of **1** were quite similar to those of the known compound 3*β*,12-dihydroxydrimenol (**1a**) [6], which suggested compound **1** possessed a drimane sesquiterpenoid skeleton. The notable difference between **1a** and **1** was that the hydroxy group at C-3 (*δ*_C_ 79.7) in **1a** was oxidized into a carbonyl group (*δ*_C_ 215.1) in **1**, which caused the downfield shifts of C-4 from *δ*_C_ 39.8 in **1a** to 47.9 in **1**, and C-2 from *δ* 28.1 in **1a** to 38.7 in **1**. The above assignment was further supported by the HMBC correlations from *δ*_H_ 1.08 (Me-14, s) and 1.02 (Me-15, s) to *δ*_C_ 215.1 (s, C-3) (Fig. 2), as well as IR absorption band at 1701 cm^−1^ and mass data analysis. In the ROESY spectrum (Fig. 2), cross peaks between Me-15/H-5, H-5/H-9, Me-13/H-11 were observed, which suggested that both Me-13 and H-11 should be *β* oriented. Therefore, compound **1** was established as 12-hydroxy-3-oxodrimenol (**1**).

**Table 1:** ^1^H NMR (600 MHz) and ^13^C NMR (150 MHz) data of **1** and **2** (*δ* in ppm, *J* in Hz)

No.	1		1a^a^	2		2a^b^
	*δ*_H_^c^	*δ*_C_^c^	*δ*_C_^d^	*δ*_H_^d^	*δ*_C_^d^	*δ*_H_^d^
1	2.76, ddd (14.5, 14.5, 5.3)	35.1, t	38.8	1.62, overlapped	27.4, t	27.3
	2.14, ddd (14.5, 3.7, 3.7)			1.67, overlapped		
2	2.34, ddd (13.3, 5.3, 3.7)	38.7, t	28.1	1.99, overlapped	37.7, t	37.6
	1.59, ddd (14.5, 13.3, 3.7)			1.28, m		
3		215.1, s	79.5	3.26, dd (11.3, 3.2)	78.9, d	78.9
4		47.9, s	39.8		38.8, s	38.7
5	1.63, dd (11.8, 4.6)	51.8, d	50.7	1.22, dd (11.8, 4.8)	49.4, d	49.3
6	2.14, overlapped	24.4, t	24.3	1.96, m	23.3, t	23.2
	2.01, m			2.01, overlapped		
7	5.78, d (5.1)	125.0, d	126.4	5.52, m	124.1, d	123.5
8		139.3, s	138.4		131.8, s	132.3
9	2.14, overlapped	54.7, d	55.8	2.05, m	53.3, d	53.2
10		36.2, s	36.6		35.9, s	35.7
11	3.70, ddd (11.1, 5.0, 2.5)	60.9, t	61.2	4.25, dd (11.5, 6.3)	64.3, t	63.0
	3.90, ddd (11.1, 6.9, 5.0)			4.39, dd (11.5, 3.3)		
12	3.96, dd (12.2, 6.9)	66.7, t	67.0	1.65, s	21.7, q	21.5
	4.27, dd (12.2, 5.3)					
13	1.04, s	14.4, q	15.0	0.81, s	14.7, q	14.5
14	1.08, s	22.5, q	15.9	0.87, s	15.4, q	15.2
15	1.02, s	25.7, q	28.7	0.98, s	28.2, q	28.0
16					173.5, s	171.2
17				4.13, dd (12.7, 5.4)	60.9, t	21.3
				4.14, dd (12.7, 5.4)		
11-OH	4.34, dd (5.0, 5.0)					
12-OH	4.20, dd (6.9, 5.3)					
17-OH				2.36, dd (5.4, 5.4)		

^a^Literature data

^b^Experimental data

^c^Spectra were measured in acetone-*d*_6_

^d^Spectra were measured in CDCl_3_

Compound **2** was isolated as white powder. The molecular formula was established to be C_17_H_28_O_4_ on the basis of HREIMS at *m/z* 296.1974 [M]^+^ (calcd for 296.1988, C_17_H_28_O_4_). The IR spectrum showed absorptions at 3442 cm^−1^ for hydroxy, 1725 cm^−1^ for carbonyl, and 1631 cm^−1^ for double bond functional groups. The ^1^H NMR (Table 1) spectrum showed resonances for four methyl singlets (*δ*_H_ 0.81, 0.87, 0.98, 1.65), two *O*-bearing methylene signals (*δ*_H_ 4.25, dd, *J* = 11.5,6.3 Hz; *δ*_H_ 4.39, dd, *J* = 11.5, 3.3 Hz and *δ*_H_ 4.13, dd, *J* = 12.7, 5.4 Hz; *δ*_H_ 4.14, dd, *J* = 12.7, 5.4 Hz), one *O*-bearing methane signal (*δ*_H_ 3.26, dd, *J* = 11.3, 3.2 Hz), and one olefinic proton (*δ*_H_ 5.52 m). The ^13^C NMR spectrum, along with the DEPT and HSQC spectra, classified the functionalities as a trisubstituted double bond, four methyl, five *sp*^3^ methylenes (two oxygenated), three *sp*^3^ methines (one oxygenated), two *sp*^3^ quaternary carbons, and one carbonyl (*δ*_C_ 173.5) (Table 1). These NMR spectroscopic data of **2** resembled those of a coexisting known compound acetoxydrim-7-en-3*β*-ol (**2a**) [7], except for the chemical shift of C-17 was 60.9 (CH_2_) in **2** instead of 21.3 ppm (CH_3_) in **2a**. Therefore, compound **2** was established as a hydroxy derivative of **2a**, which was also supported by the HMBC correlations from *δ*_H_ 4.13 (1H, d, *J* = 5.1 Hz) and 4.14 (1H, d, *J* = 5.1 Hz) to *δ*_C_ 173.5 (C-16, s) (Fig. 2), as well as mass data analysis. In the ROESY spectrum (Fig. 2), the cross peak of H-5/Me-15 suggested that C-14 was *β* oriented, while the cross peak of Me-15/H-3, as well as the constant coupling of H-3 (*δ*_H_ 3.26, dd, *J* = 11.3, 3.2 Hz), indicated 3-OH to be *β* oriented. Besides, the cross peaks of Me-14/H-2, H-2/Me-13, Me-13/H-11 proved that H-9 was *α* oriented and Me-13 was *β* oriented. Therefore, compound **2** was elucidated as 11-hydroxyacetoxydrim-7-en-3*β*-ol.

Compound **3**, a colorless oil, had an [M]^+^ peak at *m/z* 254.1873 in the HREIMS (cacld for 254.1882), corresponding to a molecular formula C_15_H_26_O_3_. 1D NMR spectra in combination with HSQC spectrum demonstrated 15 carbons, which were classified into three methyl, six methylenes (one olefinic carbon, one oxygenated carbon), three methines (two olefinic carbons) and three quaternary carbons (one olefinic carbon) (Table 2). All these data suggested that compound **3** was a sesquiterpenoid possessing the same skeleton with 2,6,10-trimethyl-7,10-epoxy-2,11-dodecadien-6-ol [8]. The only difference between them was that one proton of Me-15 (*δ*_C_ 24.2, q) in 2,6,10-trimethyl-7,10-epoxy-2,11-dodecadien-6-ol was replaced by a hydroxyl (*δ*_C_ 67.4, t) in **3**. This change was supported by the HMBC correlations of H-15 (*δ*_H_ 3.47, d, *J* = 11.4, 4.4 Hz, *δ*_H_ 3.42, dd, *J* = 11.4, 6.2 Hz)/C-10(*δ*_C_ 85.8, s) and H-15/C-11 (*δ*_C_ 141.1, d) (Fig. 2). In the ROESY spectrum (Fig. 2), correlations of H-15/H-9*β* (*δ*_H_ 1.97) and H-9*β*/H-7 indicated that H-15 and H-7 were in the same side. Therefore, compound **3** was identified as 2,6-dimethyl-7,10-epoxy-10-hydroxymethyldodeca-2,11-dien-6-ol.

**Table 2:** ^1^H NMR (600 MHz) and ^13^C NMR (150 MHz) data of **3** and **4** in CDCl_3_ (*δ* in ppm, *J* in Hz)

No.	3		4	
	*δ*_C_	*δ*_H_	*δ*_C_	*δ*_H_
1	17.8, q	1.62, s	18.4, q	1.68, s
2	131.9, s		136.6, s	
3	124.5, d	5.04, br. t (7.3)	125.9, d	5.11, br. d (8.4)
4	22.3, t	2.10, m	73.7, d	4.77, ddd (10.6, 8.4, 6.4)
		2.03, overlapped		
5	37.5, t	1.51, ddd (13.7, 11.6, 5.2)	47.8, t	2.06, dd (13.1, 6.4)
		1.35, ddd (13.7, 11.6, 5.4)		1.61, overlapped
6	72.9, s		73.1, s	
7	86.9, d	3.81, dd (9.0, 6.0)	87.9, d	3.59, dd (10.3, 3.2)
8	25.9, t	1.81, overlapped	26.5, t	1.59, overlapped
		1.78, m		1.33, m
9	32.6, t	1.97, overlapped	39.1, t	1.81, ddd (14.9, 11.1, 5.6)
		1.83, overlapped		1.62, overlapped
10	85.8, s		80.3, s	
11	141.1, d	5.83, dd (17.5, 10.9)	145.2, d	5.90, dd (17.5, 10.9)
12	115.0, t	5.23, dd (17.5, 1.1)	111.8, t	5.22, dd (17.5, 1.1)
		5.12, dd (10.9, 1.1)		5.05, dd (10.9, 1.1)
13	25.9, q	1.68, s	26.0, q	1.72, s
14	24.4, q	1.25, s	28.2, q	1.29, s
15	67.4, t	3.47, dd (11.4, 4.4)	23.7, q	1.29, s
		3.42, dd (11.4, 6.2)		
15-OH		3.43, dd (6.2, 4.4)		

Compound **4** was isolated as a colorless oil. Its molecular formula was determined as C_15_H_26_O_3_ based on the HRESIMS at *m/z* 277.1772 (cacld for 277.1780, [M + Na]^+^). Its NMR data were similar to those of 2,6,10-trimethyl-7,10-epoxy-2,11-dodecadien-6-ol [8]. Compared with compound **3**, one proton at C-4 of compound **4** was substituted by a hydroxy, while Me-15 of **4** was not oxygenated. This change was supported by ^1^H-^1^H COSY correlation of H-4/H-5 and HMBC correlation of H-4/C-2 (Fig. 2). The ROESY data suggested the other parts of **4** were same to those of **3** (Fig. 2). Therefore, the structure of compound **4** was elucidated as 7,10-epoxy-2,6,10-trimethyldodeca-2,11-diene-4,6-diol.

Fourteen known compounds were identified as 3,11-dihydrodrimane [9], 3*β*,11,12-trihydroxy-drimene (**1a**), 3*β*-hydroxydrimenol, 11,12-dihydroxydrimene [6], acetoxydrim-7-en-3*β*-ol (**2a**) [7], 3-keto-drimenol [10], drimane-2,11-diol [11], 11-hydroxydrim-8-en-7-one [12], (−)-drimenol [13], 15-hydroxy-T-muurolol [14], 2*β*-hydroxy-*δ*-cadinol [15], 2*β*-hydroxy-*α*-cadinol [16], 3*β*-hydroxy-*δ*-cadinol [17], and 2-(5′-ethenyltetrahydro-5′-methylfuran-2′-yl)-6-methylhept-5-en-ol [18] by comparison of spectroscopic data with those reported in the literature.

Unfortunately, none of them showed significant inhibitory activities against the five human cancer cell lines (> 40 *μ*mol).

## Experimental

### General Experimental and Procedures

Optical rotations were obtained on a JASCO P-1020 digital polarimeter. IR and UV spectra were recorded on a Bruker Tensor 27 FT-IR spectrometer with KBr pellets and a Shimadzu UV-2401PC instrument, respectively. 1D and 2D NMR spectra were obtained on a Bruker Avance III 600 MHz spectrometer. ESIMS and HREIMS were measured on a Waters Xevo TQ-S spectrometer and a Waters Autospec Premier P776 spectrometer, respectively. HRESIMS was measured on an Agilent G6230 TOF MS spectrometer. Silica gel 200–300 mesh (Qingdao Marine Chemical Inc., China) and Sephadex LH-20 (Amersham Biosciences, Sweden) were used for column chromatography. Medium pressure liquid chromatography (MPLC) was performed on a Büchi Sepacore System equipping pump manager C-615, pump modules C-605 and fraction collector C-660 (Büchi Labortechnik AG, Switzerland), and columns packed with Chromatorex C-18 (40–75 *μ*m, Fuji Silysia Chemical Ltd., Japan). Preparative high performance liquid chromatography (Prep-HPLC) was performed on an Agilent 1260 liquid chromatography system equipped with a Zorbax SB-C18 column (5 *μ*m, 9.4 × 150 mm).

### Fungus Material

The fungus *Phellinidium sulphurascens* was collected from Changbai Mountain, Jilin Province, China in 2009, and were identified by Prof. Yu-Cheng Dai (Institute of Microbiology, Beijing Forestry University). The culture medium to ferment this fungus consist of glucose (5 %), peptone from porcine meat (0.15 %), yeast powder (0.5 %), KH_2_PO_4_ (0.05 %) and MgSO_4_ (0.05 %). Five hundred 500-mL Erlenmeyer flasks each containing 350 mL of above-mentioned culture medium were inoculated with *P. sulphurascens* strains, respectively. Then they were incubated on rotary shakers at 24 °C and 150 rpm for 25 days in dark environment.

### Extraction and Isolation

The culture broth (20 L) of *P. sulphurascens* was filtered, the filtrate was extracted four times with ethyl acetate (EtOAc). Meanwhile, the mycelium was extracted by CHCl_3_/MeOH (1:1) for three times. The EtOAc layer together with the mycelium extraction was concentrated under reduced pressure to afford a crude extract (6.0 g). Then this residue was decolorized by Sephadex LH-20 column chromatography (CHCl_3_:MeOH = 1:1), followed by MPLC eluting with MeOH/H_2_O (from 20:80 to 100:0) to give seven main fractions (A–G). Fraction C (0.8 g) was separated by Sephadex LH-20 column chromatography (MeOH) to afford three subfractions (C1–C3). Subfraction C2 was subjected to Sephadex LH-20 column chromatography (acetone) to afford six fractions (C2a–C2c). Fraction C2c was separated on a Prep-HPLC to afford **1** (1.2 mg), **1a** (3.8 mg), 3*β*-hydroxydrimenol (5.3 mg). A similar purification procedure on fraction D (1.1 g) yielded **2** (1.5 mg), **2a** (2.8 mg), 11,12-dihydroxydrimene (5.8 mg), 3-keto-drimenol (3.3 mg), while fraction E yielded **3** (0.8 mg), **4** (0.9 mg), drimene-2,11-diol (7.2 mg), 11-hydroxydrim-8-en-7-one (6.8 mg), 3,11-dihydrodrimane (4.0 mg), (−)-drimenol (4.5 mg), 15-hydroxy-T-muurolol (3.2 mg). 2*β*-hydroxy-*δ*-cadinol (4.5 mg), 2*β*-hydroxy-*α*-cadinol (4.4 mg), 3*β*-hydroxy-*δ*-cadinol (4.3 mg), and 2-(5′-ethenyltetrahydro-5′-methylfuran-2′-yl)-6-methylhept-5-en-ol (7.9 mg).

#### 12-Hydroxy-3-oxodrimenol (**1**)

Amorphous powder, ${\left[\alpha \right]}_{\text{D}}^{18}$ −56.9 (*c* = 0.03, MeOH). IR (KBr) ν_max_ cm^−1^: 3440, 2971, 2933, 1701, 1630, 157, 1426, 1384, 1033. For ^1^H NMR (600 MHz, acetone-*d*_6_) and ^13^C NMR (150 MHz, acetone-*d*_6_) spectroscopic data, see Table 1. HREIMS *m*/*z*: 252.1723 [M]^+^ (calcd for C_15_H_24_O_3_, 252.1725).

#### 11-Hydroxyacetoxydrim-7-en-3β-ol (**2**)

Amorphous powder, ${\left[\alpha \right]}_{\text{D}}^{18}$ +6.2 (*c*0.03, MeOH). IR (KBr) ν_max_ cm^−1^: 3442, 2958, 2923, 2854, 1725, 1631, 1448, 1428, 1383, 1104, 1034. ^1^H NMR (600 MHz, CDCl_3_) and ^13^C NMR (150 MHz, CDCl_3_) spectroscopic data, see Table 1. HREIMS *m*/*z*: 296.1974 [M]^+^ (calcd for C_17_H_28_O_4_, 296.1988).

#### 2,6-Dimethyl-7,10-epoxy-10-hydroxymethyldodeca-2,11-dien-6-ol (**3**)

Colorless oil, ${\left[\alpha \right]}_{\text{D}}^{18}$ +5.9 (*c*0.03, MeOH). IR (KBr) ν_max_ cm^−1^: 3445, 2957, 2922, 2852, 1634, 1384, 1099, 1036. ^1^H NMR (600 MHz,CDCl_3_) and ^13^C NMR (150 MHz, CDCl_3_) spectroscopic data, see Table 2. HREIMS *m*/*z*: 254.1873 [M]^+^ (calcd for C_15_H_26_O_3_, 254.1882).

#### 7,10-Epoxy-2,6,10-trimethyldodeca-2,11-diene-4,6-diol (**4**)

Colorless oil, ${\left[\alpha \right]}_{\text{D}}^{18}$ +0.4 (*c* = 0.01, MeOH). IR (KBr) ν_max_ cm^−1^: 3432, 2957, 2923, 2853, 1630, 1463, 1384, 1111, 1038. ^1^H NMR (600 MHz, CDCl_3_) and ^13^C NMR (150 MHz, CDCl_3_) spectroscopic data, see Table 2. HRESIMS *m*/*z*: 277.1772 [M+Na]^+^ (calcd for C_15_H_26_NaO_3_, 277.1780).

### Cytotoxicity

The cytotoxicity assay was performed according to the MTT method in 96-well microplates. Five human cancer cell lines: human myeloid leukemia HL-60, hepatocellular carcinoma SMMC-7721, lung cancer A-549, breast cancer MCF-7 and human colon cancer SW480 cells were used in the cytotoxicity assay. All the cells were cultured in RPMI-1640 or DMEM (Hyclone, Logan, UT, USA), supplemented with 10 % fetal bovine serum (Hyclone) in 5 % CO_2_ at 37 °C. Briefly, 100 *μ*L of adherent cells were seeded into each well of 96-well cell culture plates and allowed to adhere for 12 h before drug addition, while suspended cells were seeded just before drug addition with an initial density of 1 × 10^5^ cells/mL. Each tumor cell line was exposed to the test compound at concentrations of 0.064, 0.32, 1.6, 8, and 40 *μ*mol in triplicates for 48 h, with cisplatin (sigma, USA) as a positive control (IC_50_: SW480, 12.0 *μ*mol; SMMC-7721, 10.2 *μ*mol; HL-60, 3.1 *μ*mol; MCF-7, 17.5 *μ*mol; A-549, 9.1 *μ*mol). After compound treatment, cell viability was detected and cell growth curve was graphed. 

## Electronic supplementary material

Below is the link to the electronic supplementary material.

Supplementary material 1 (DOCX 6316 kb)

## References

[1] Dai YC (2003). Chin. J. Appl. Ecol..

[2] Wu X, Lin S, Zhu C, Yue Z, Yu Y, Zhao F, Liu B, Dai J, Shi J (2010). J. Nat. Prod..

[3] Song AR, Sun XL, Kong C, Zhao C, Qin D, Huang F, Yang S (2014). Arch. Virol..

[4] Wang J, Hu F, Luo Y, Luo H, Huang N, Cheng F, Deng Z, Deng W, Zou K (2014). Fitoterapia.

[5] Pei JJ, Wang ZB, Ma HL, Yan JK (2015). Carbohyd. Polym..

[6] Aranda G, Facon I, Lallemand JY, Leclaire M, Azerad R, Cortes M, Lopez J, Ramirez H (1992). Tetrahedron Lett..

[7] Ramirez HE, Cortes MM, Agosin E (1993). J. Nat. Prod..

[8] Markovic I, Djarmati Z, Abramovic B (2008). J. Serb. Chem. Soc..

[9] Mori K, Komatsu M (1987). Tetrahedron.

[10] Xu D, Yu S, Zhou ZY, Liu R, Leng Y, Liu JK (2009). Chem. Pharm. Bull..

[11] Falk R, Timm A, Olov S (2002). Tetrahedron.

[12] Panasenko AA, Gorincioi EC, Aricu AN, Barcari EA, Deleanu K, Lad PF (2004). Russ. Chem. Bull. Int. Ed..

[13] Manuel C, Virginia D, Claudio S, Veronica A (2011). Nat. Prod. Commun..

[14] Kuo YH, Chen CH, Chien SC, Lin YL (2002). J. Nat. Prod..

[15] Lin YT, Cheng YS, Kuo YH, Lin KC (1974). J. Chin. Chem. Soc..

[16] Teresa JP, Alle MAVM, Gonzalez MS, Bellido IS (1984). Tetrahedron.

[17] Tsypysheva IP, Kunakova AM, Valeev FA, Tolstikov GA (2001). Chem. Nat. Compd..

[18] Holmes DS, Ashworth DM, Robinson JA (1990). Helv. Chim. Acta.

