# Supplementary material for: Four New Sesquiterpenoids from Cultures of the Fungus Phellinidium sulphurascens
Source: Nat Prod Bioprospect. 2014 Dec 10;5(1):23–8. doi: 10.1007/s13659-014-0047-x (PMC4328000; doi:10.1007/s13659-014-0047-x)
Supplement: Supplementary file 1 — Supplementary material 1 (DOCX 6316 kb) [file 13659_2014_47_MOESM1_ESM.docx]

Supporting information

**Four New Sesquiterpenoids from Cultures of the Fungus *Phellinidium sulphurascens***

Zhen-Zhu Zhao^a,b^, He-Ping Chen^a,b^, Tao Feng^a^, Zheng-Hui Li^a^, Ze-Jun Dong^a^ and Ji-Kai Liu^a,^^[[1]](#footnote-2)^*

*^a^ State Key Laboratory of Phytochemistry and Plant Resources in West China, Kunming Institute of Botany, Chinese Academy of Sciences, Kunming 650201, People’s Republic of China;*

*^b^University of Chinese Academy of Sciences, Beijing 100049, People’s Republic of China*

**Figure 1S-7S**. NMR and mass spectra ofcompound **1**.

**Figure 8S-14S**. NMR and mass spectra of compound **2**.

**Figure 15S-16S**. NMR spectra of compound **2a**.

**Figure 17S-23S**. NMR and mass spectra of compound **3**.

**Figure 24S-30S**. NMR and mass spectra of compound **4**.

**Figure 1S.** ^1^H NMR spectrum of compound **1** (600MHz, acetone-*d*_6_).


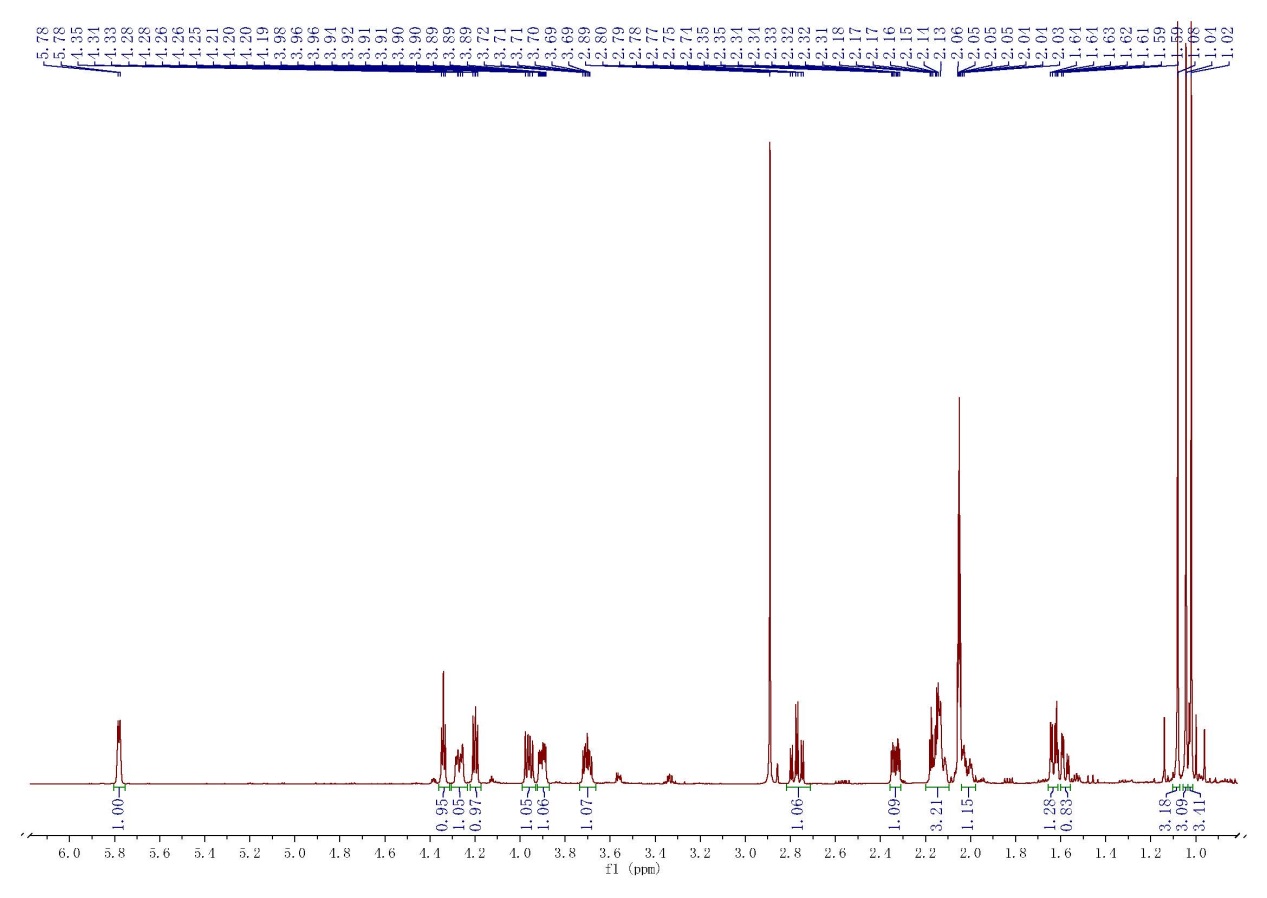

**Figure 2S.** ^13^C NMR and DEPT spectrum of compound **1** (150MHz, acetone-*d*_6_).

**Figure 3S**. HSQC spectrum of compound **1** (600MHz, acetone-*d*_6_).


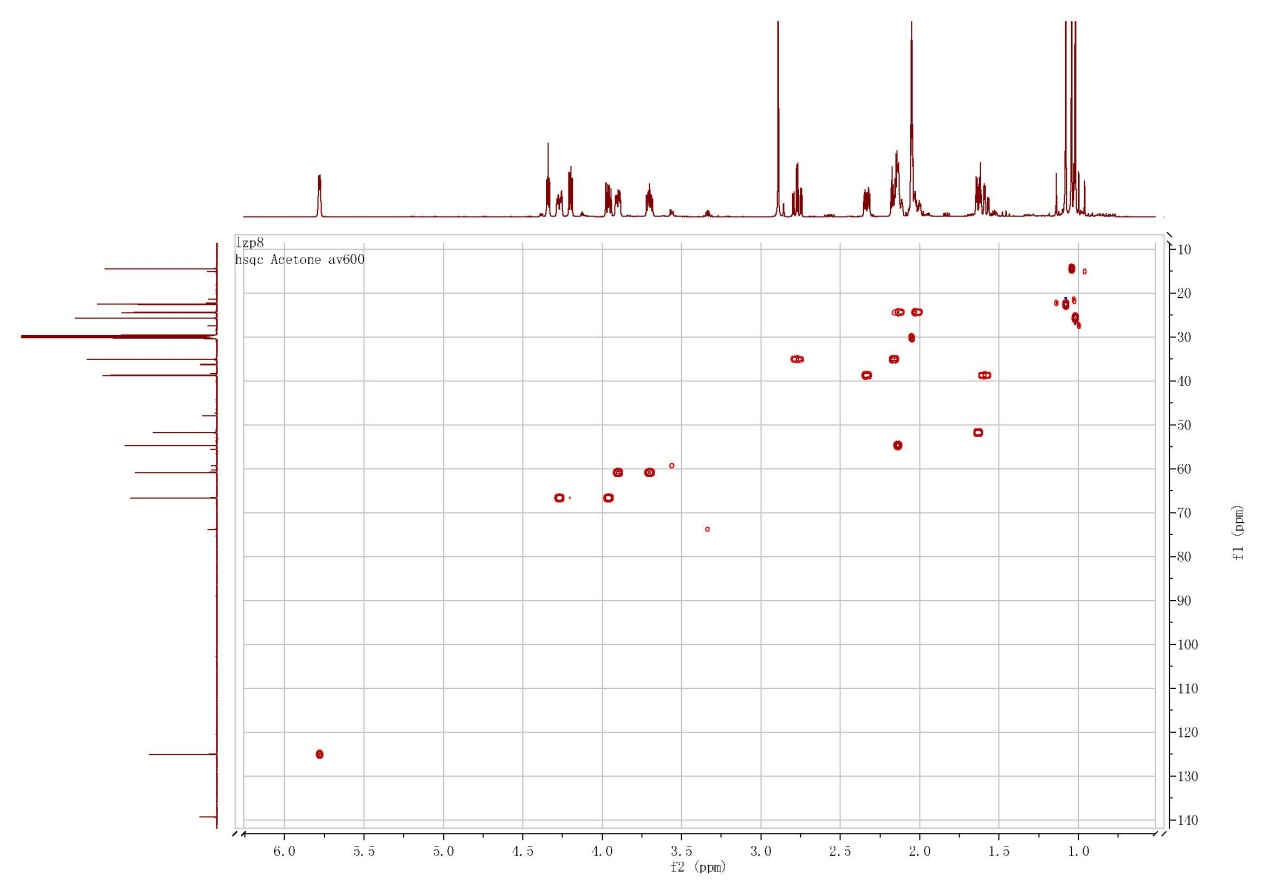

**Figure 4S**. HMBC spectrum of compound **1** (600MHz, acetone-*d*_6_).


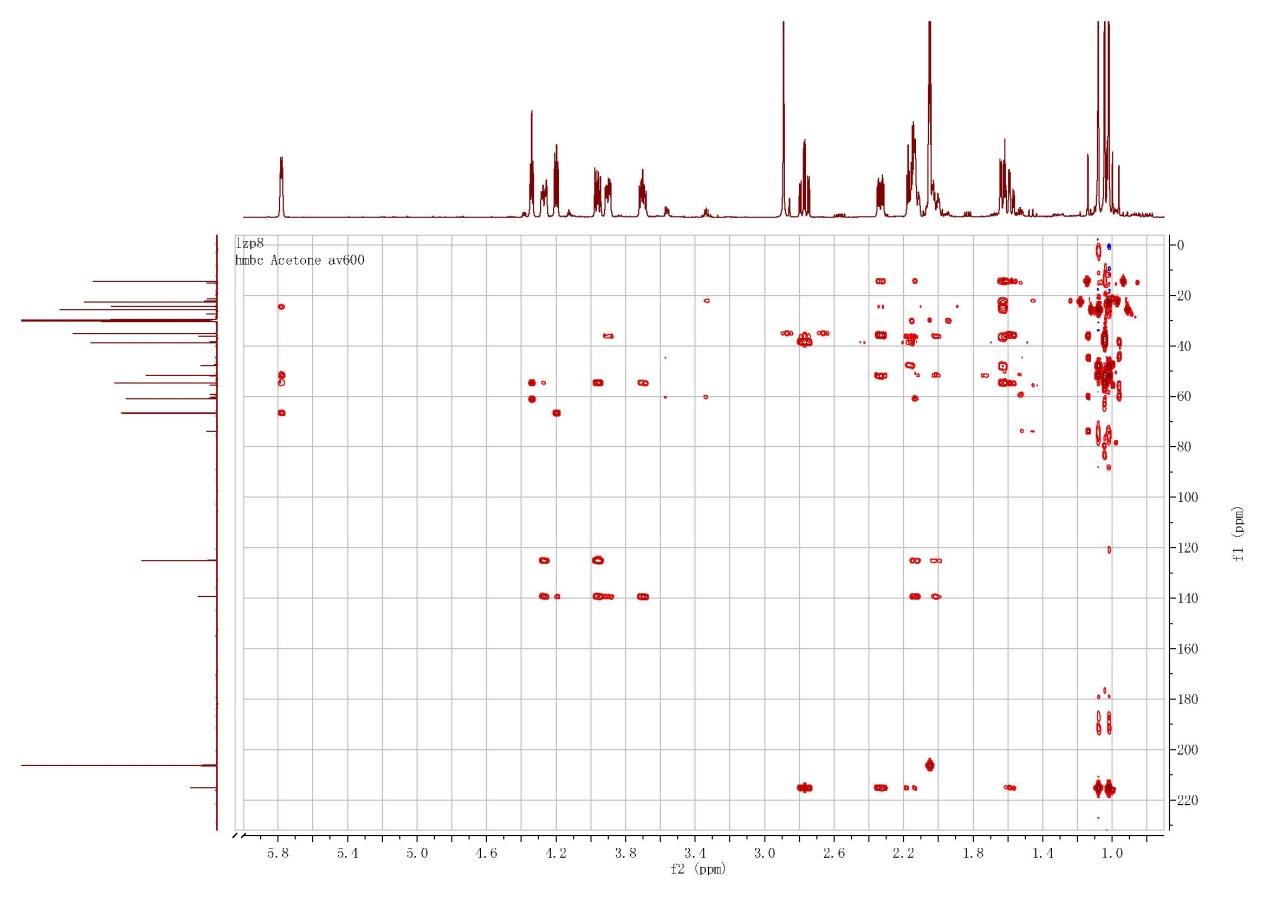

**Figure 5S.** ^1^H-^1^H COSY spectrum of compound **1** (600MHz, acetone-*d*_6_).


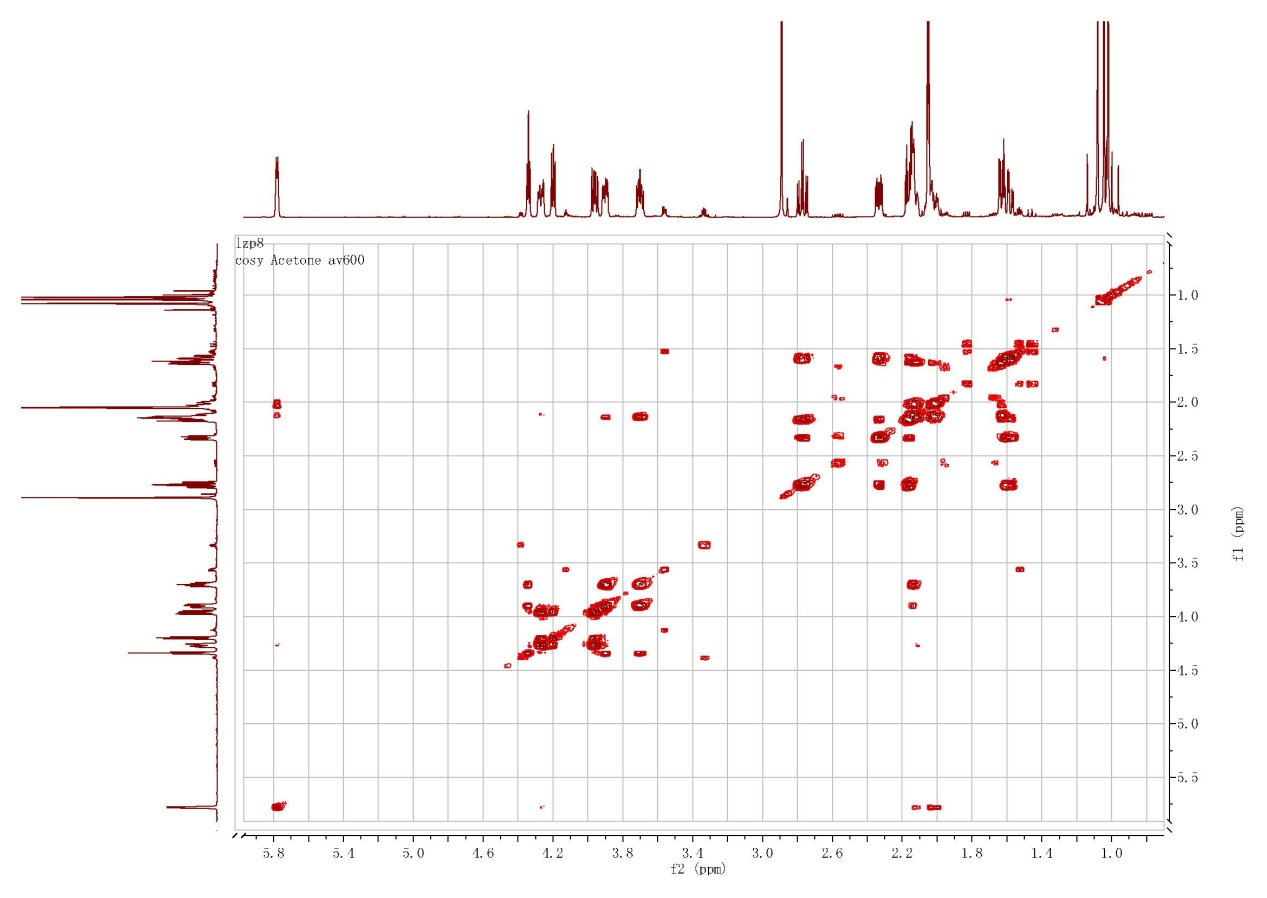

**Figure 6S**. ROESY spectrum of compound **1** (600MHz, acetone-*d*_6_).


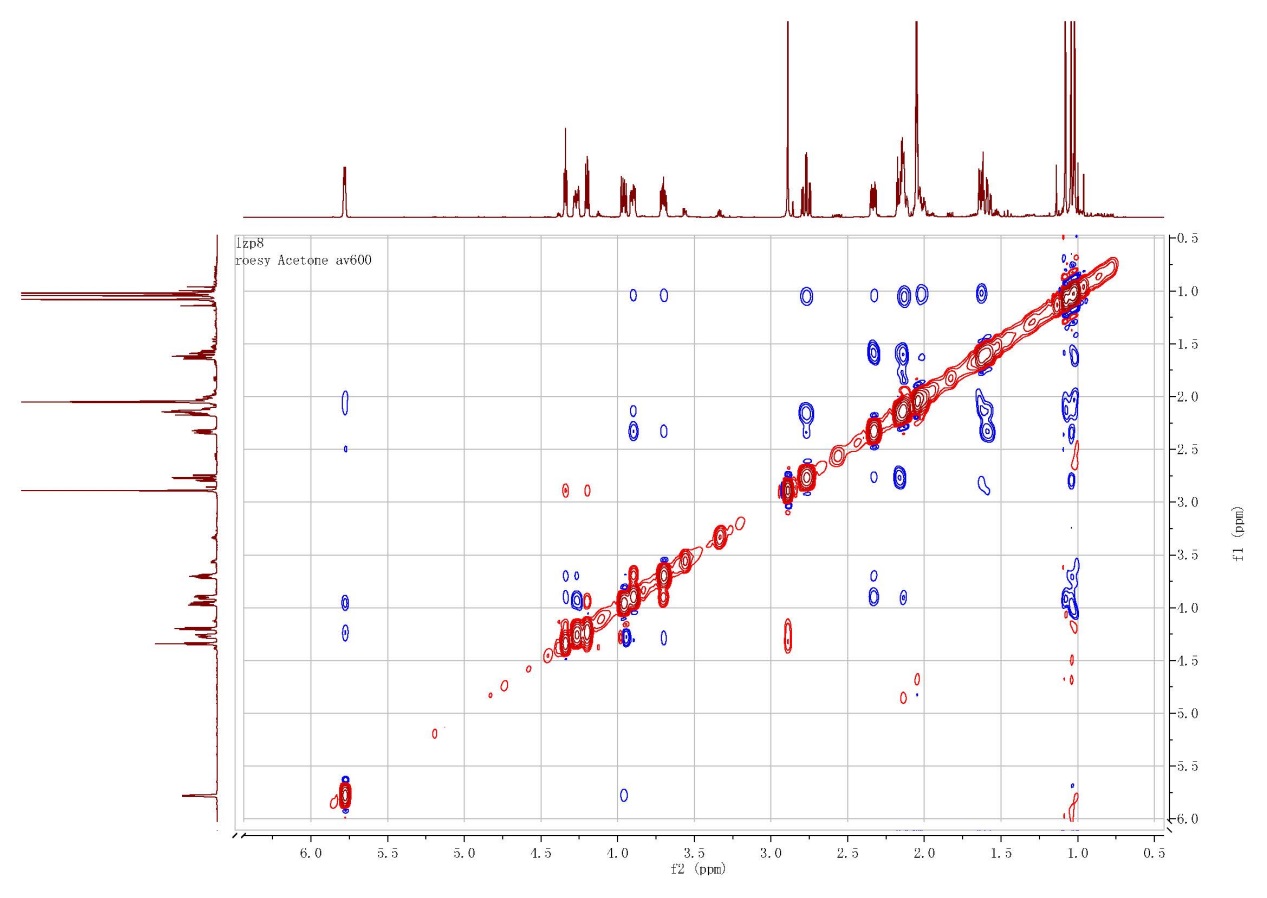

**Figure 7S**. HREIMS spectrum of compound **1**.


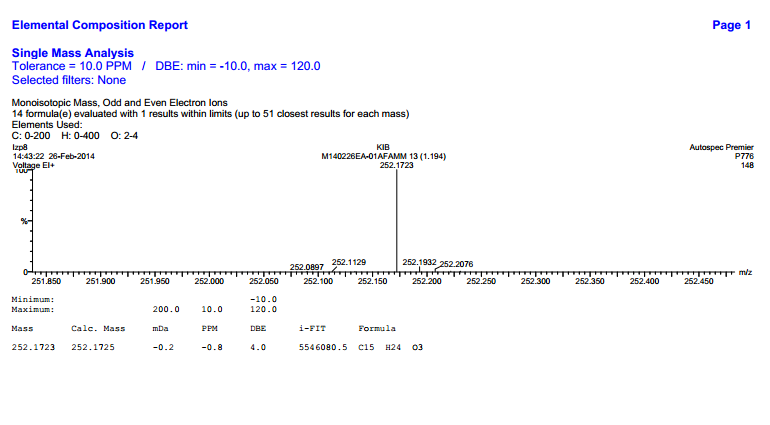


**Figure 8S.** ^1^H NMR spectrum of compound **2** (600MHz, CDCl_3_).


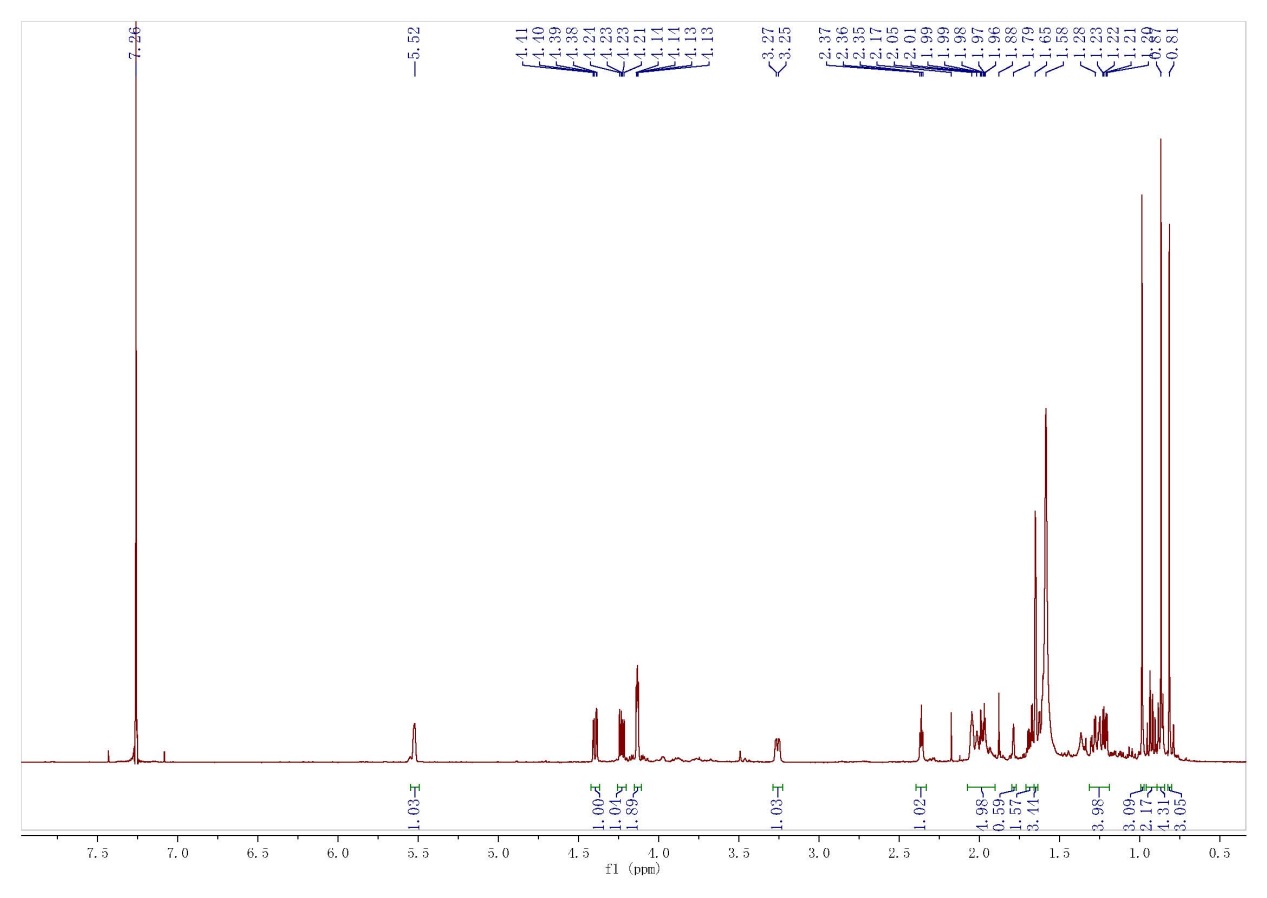

**Figure 9S.** ^13^C NMR spectrum of compound **2** (150 MHz, CDCl_3_).


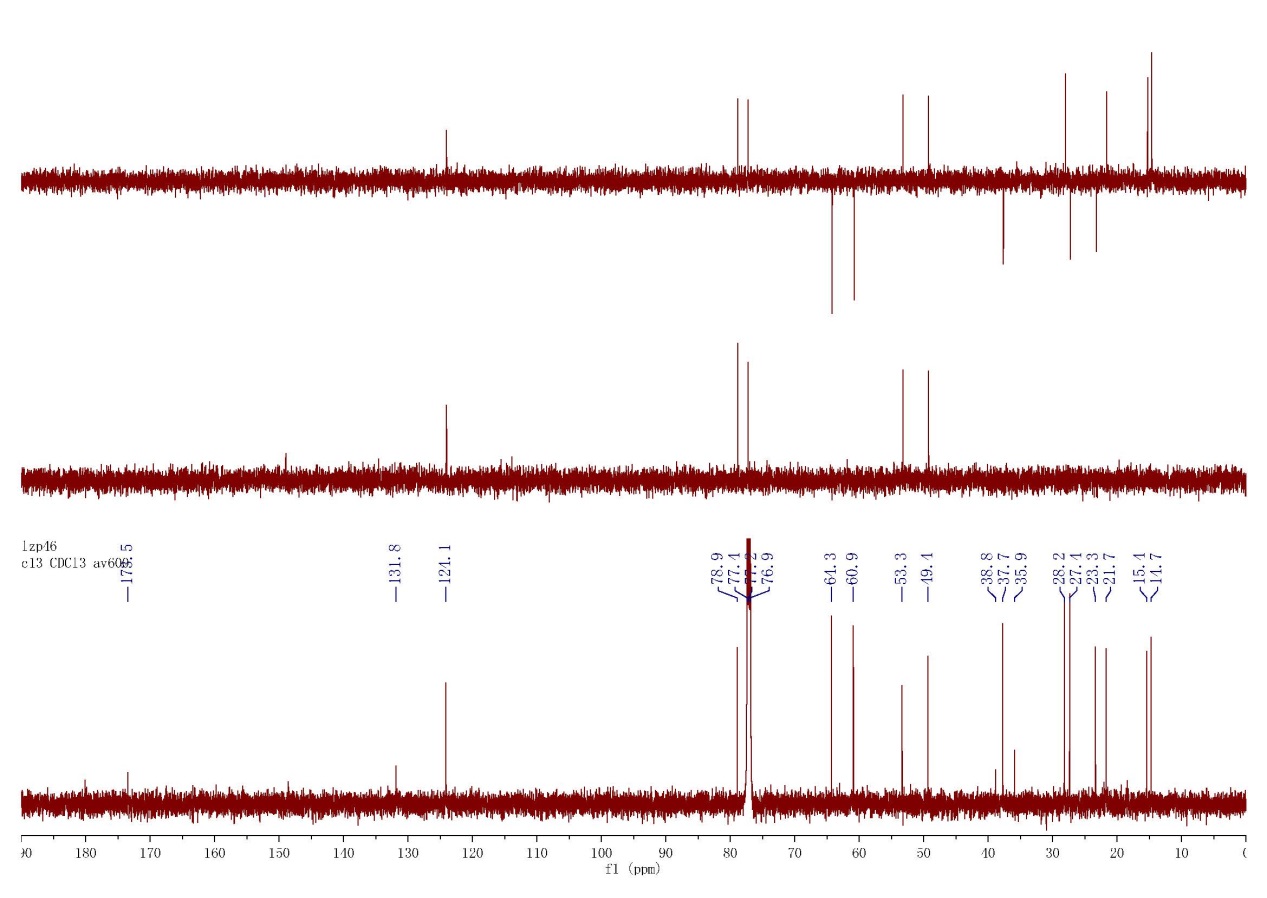

**Figure 10S.** HSQC spectrum of compound **2** (600MHz, CDCl_3_).


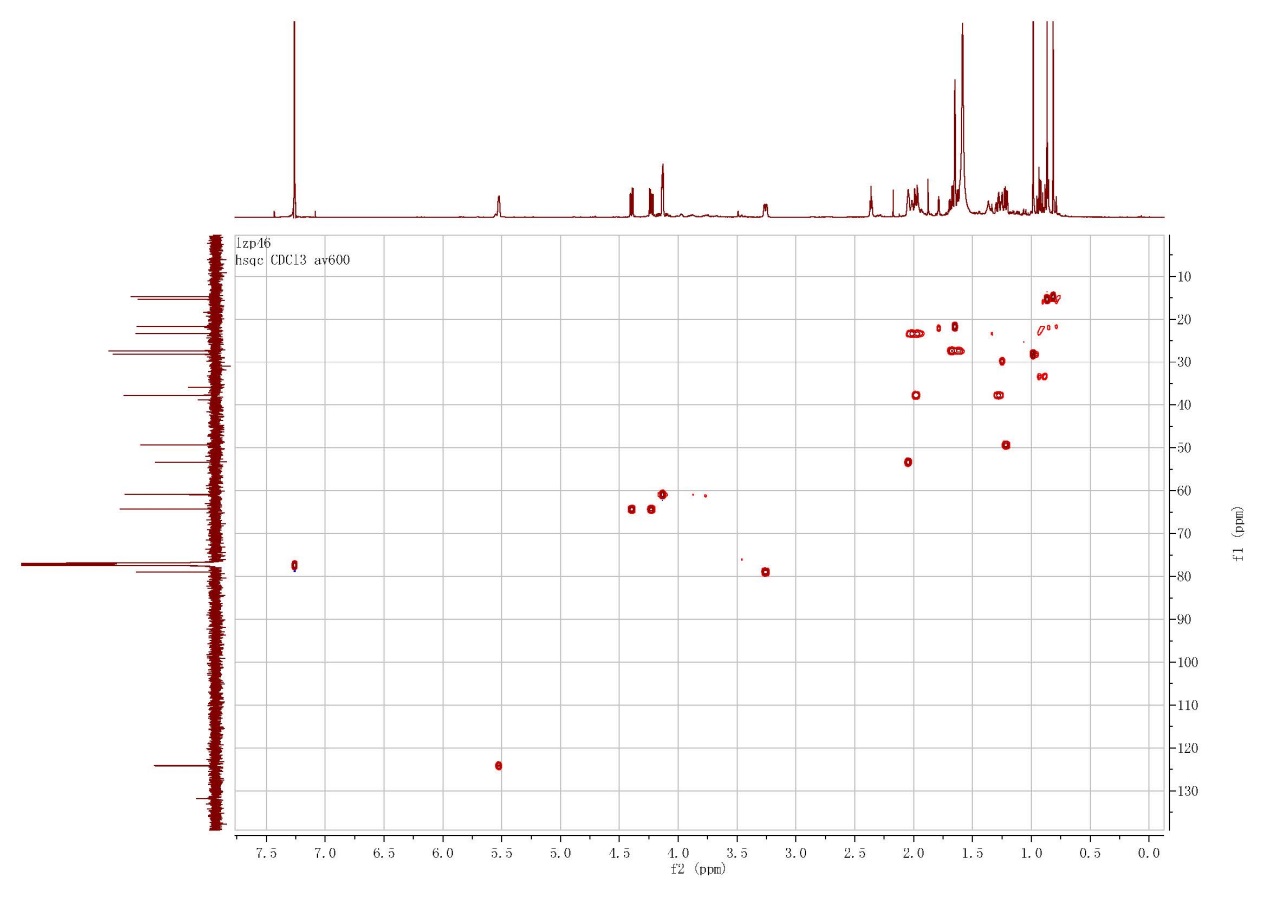

**Figure 11S.** HMBC spectrum of compound **2** (600MHz, CDCl_3_).


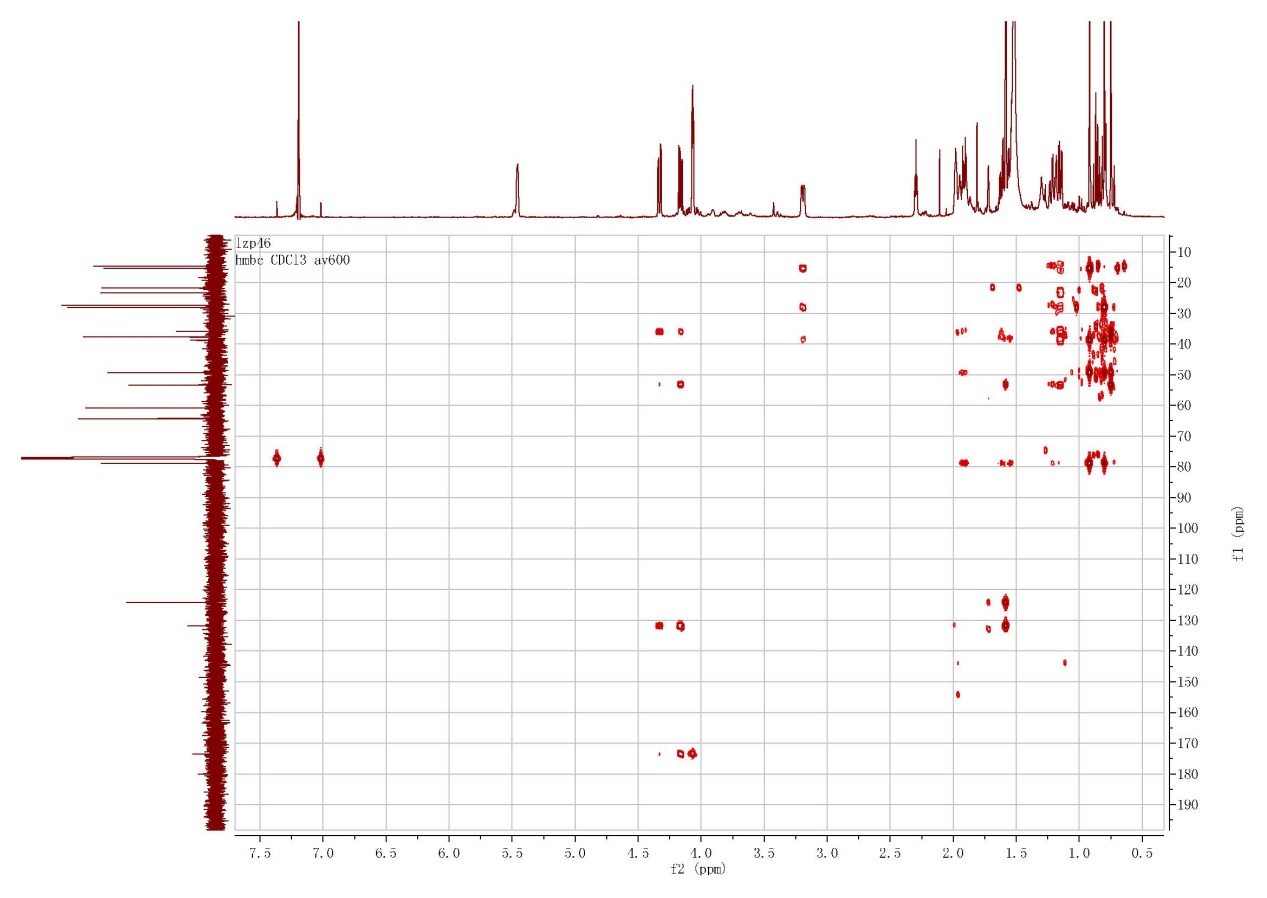

**Figure 12S.** ^1^H-^1^H COSY spectrum of compound **2** (600MHz, CDCl_3_).

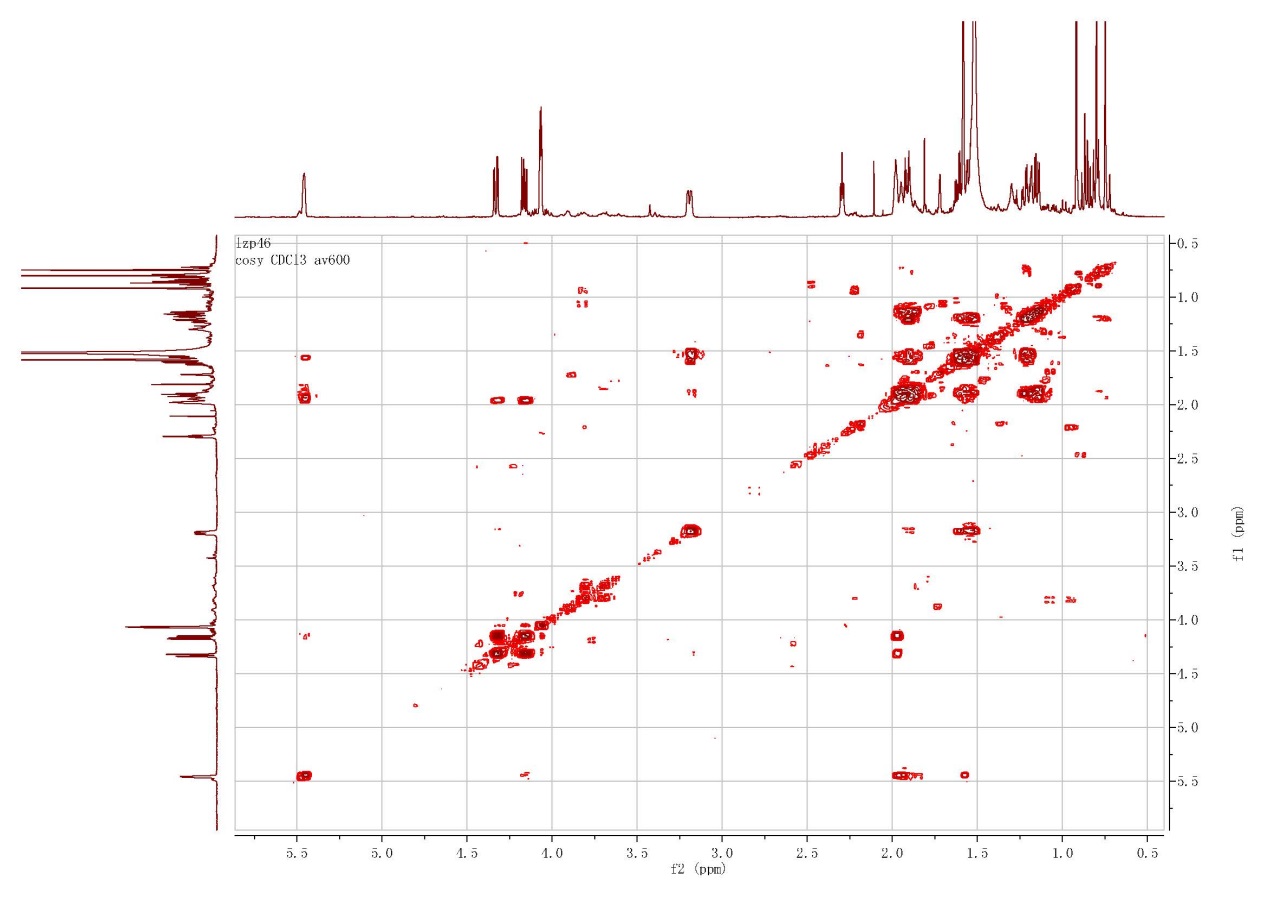


**Figure 13S.** ROESY spectrum of compound **2** (600MHz, CDCl_3_).

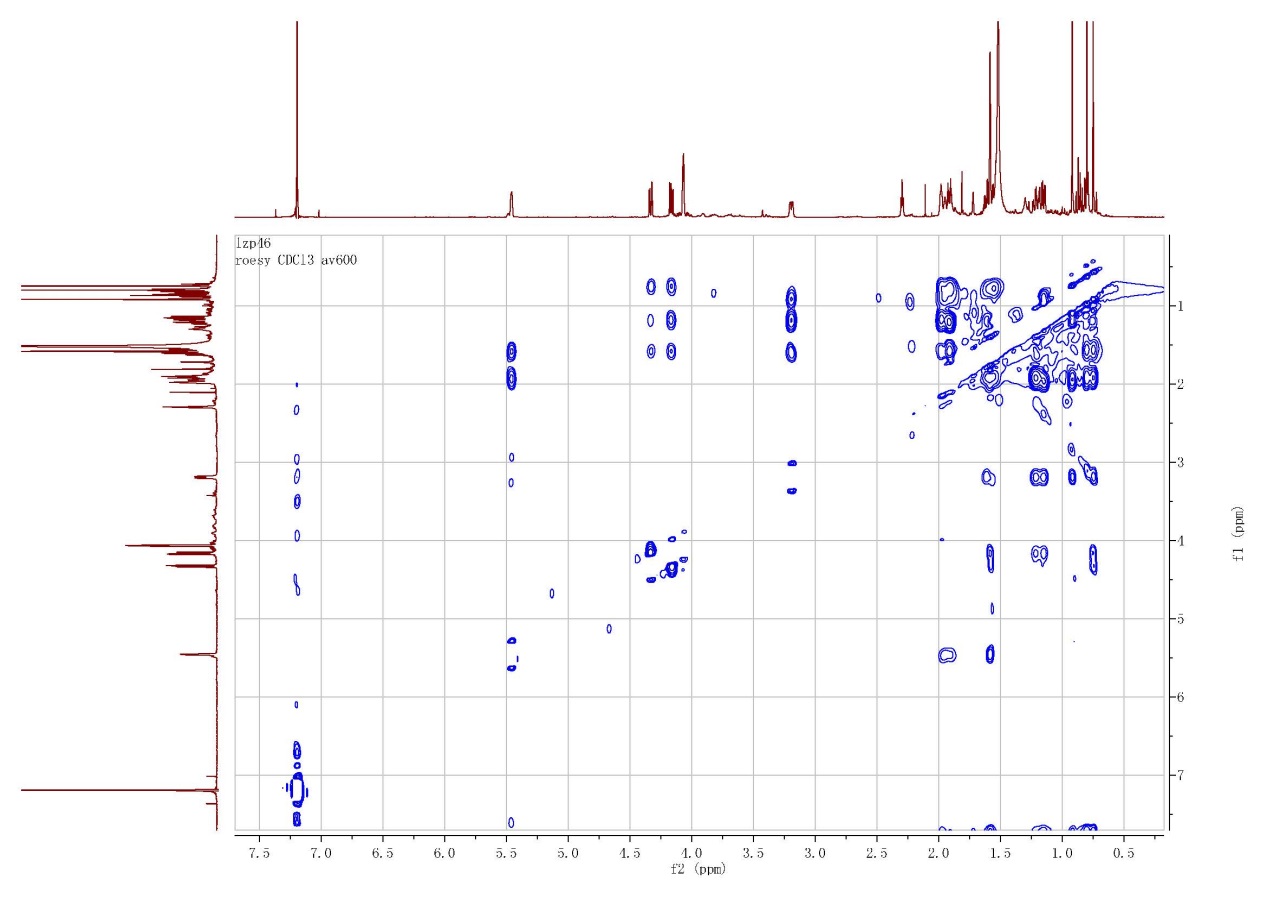


**Figure 14S**. HREIMS spectrum of compound **2**.


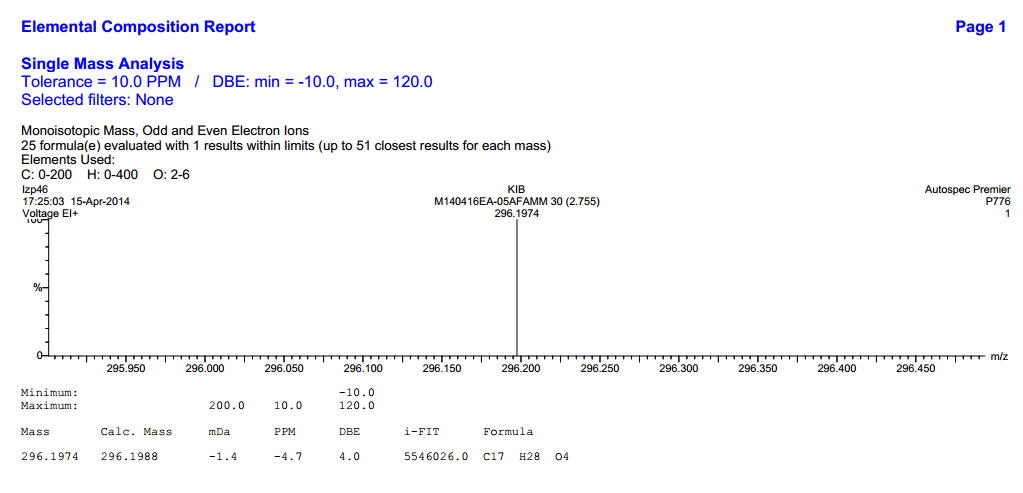


**Figure 15S.** ^1^H NMR spectrum of compound **2a** (600MHz, CDCl_3_).

**Figure 16S.** ^13^C NMR spectrum of compound **2a** (150MHz, CDCl_3_).

**Figure 17S.** ^1^HNMR spectrum of compound **3** (600MHz, CDCl_3_).


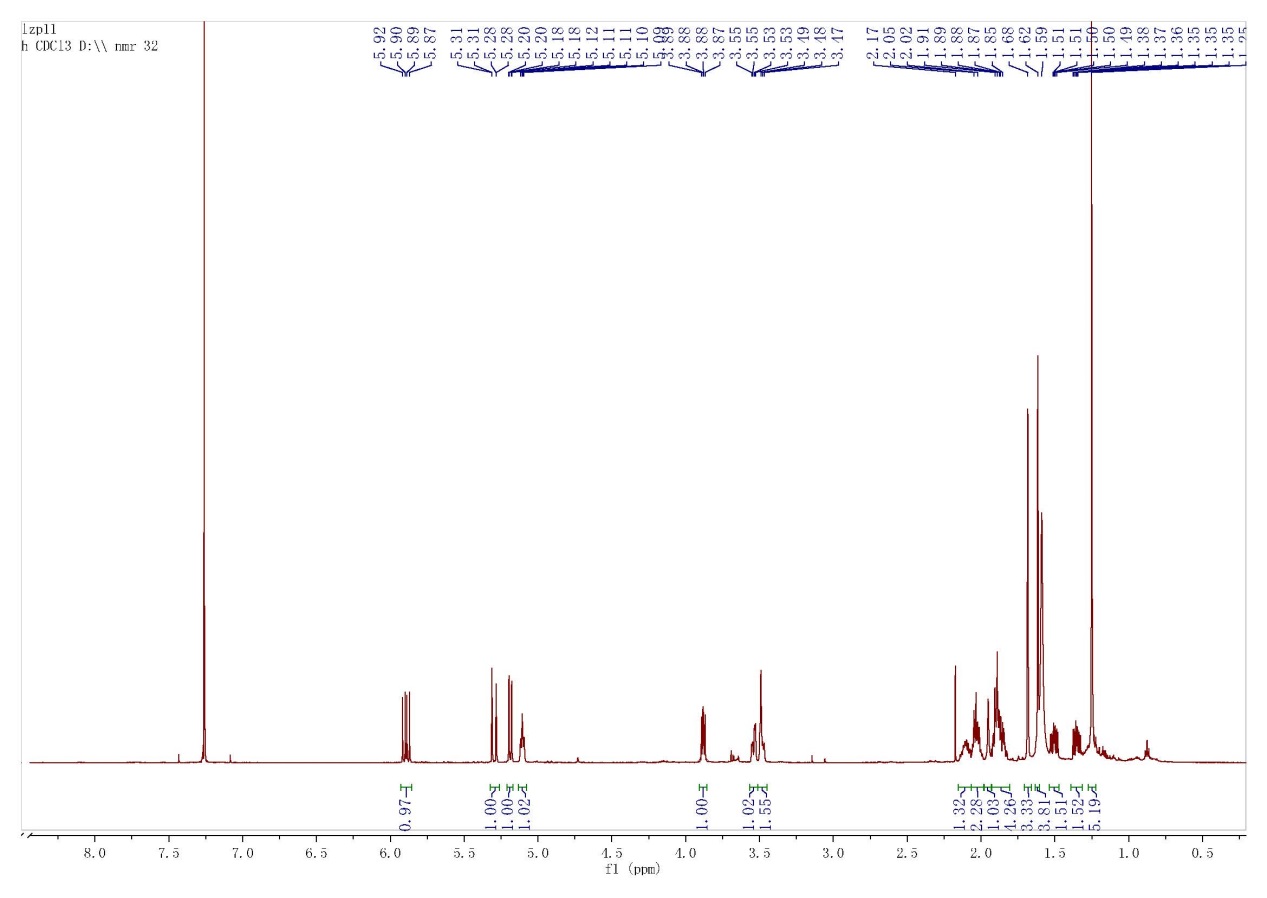

**Figure 18S.**^13^CNMR spectrum of compound **3** (150MHz, CDCl_3_).


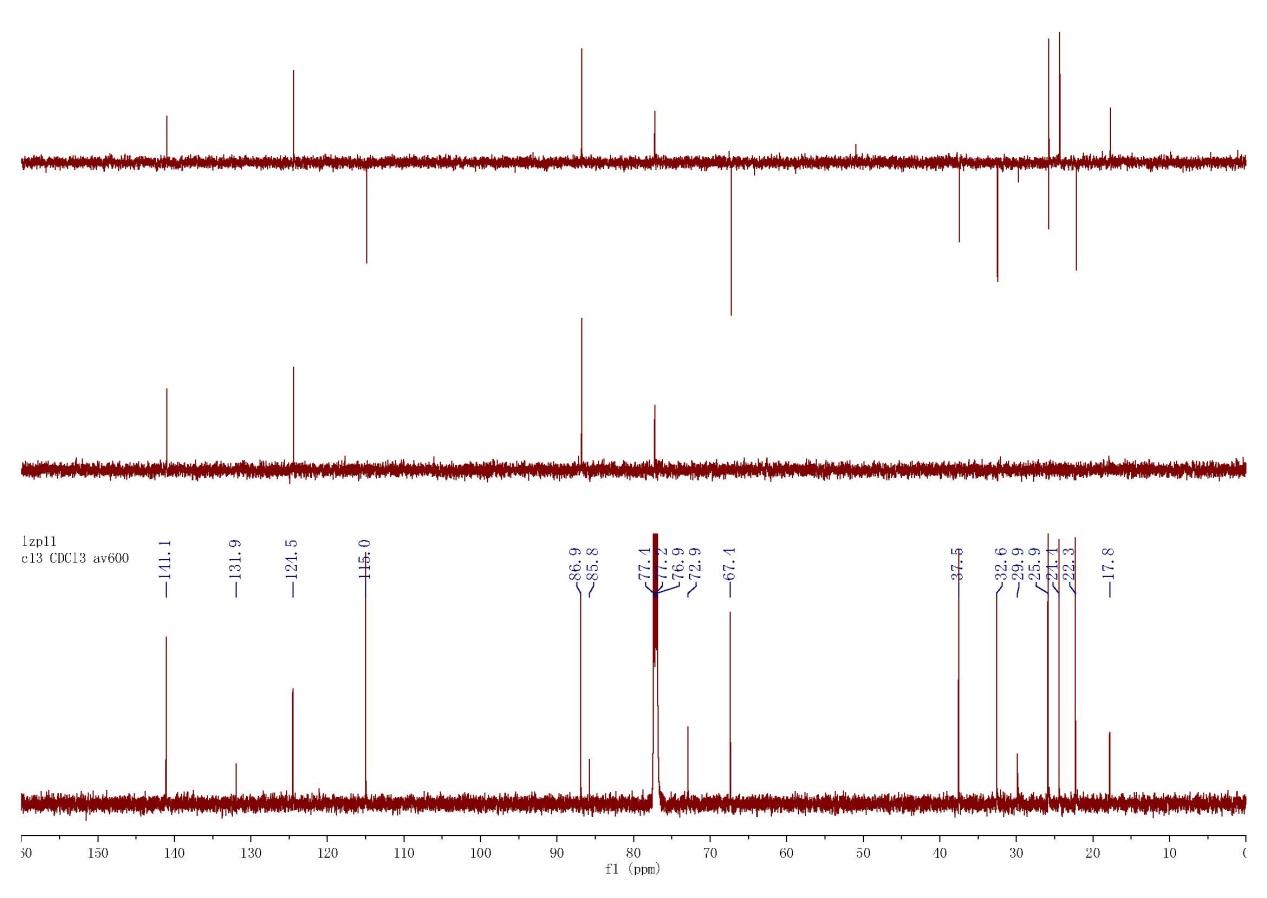

**Figure 19S.** HSQC spectrum of compound **3** (600MHz, CDCl_3_).


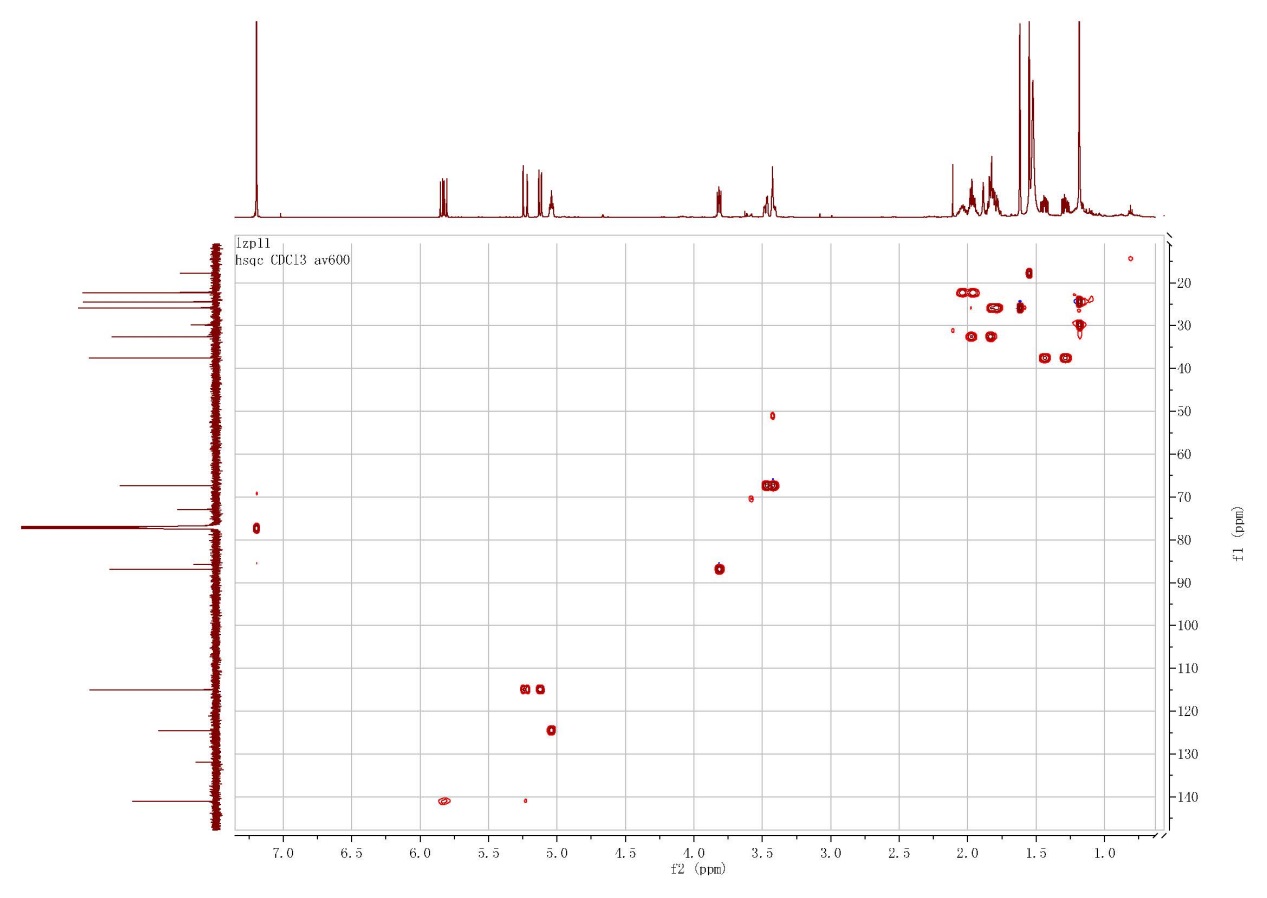

**Figure 20S.** HMBC spectrum of compound **3** (600MHz, CDCl_3_).


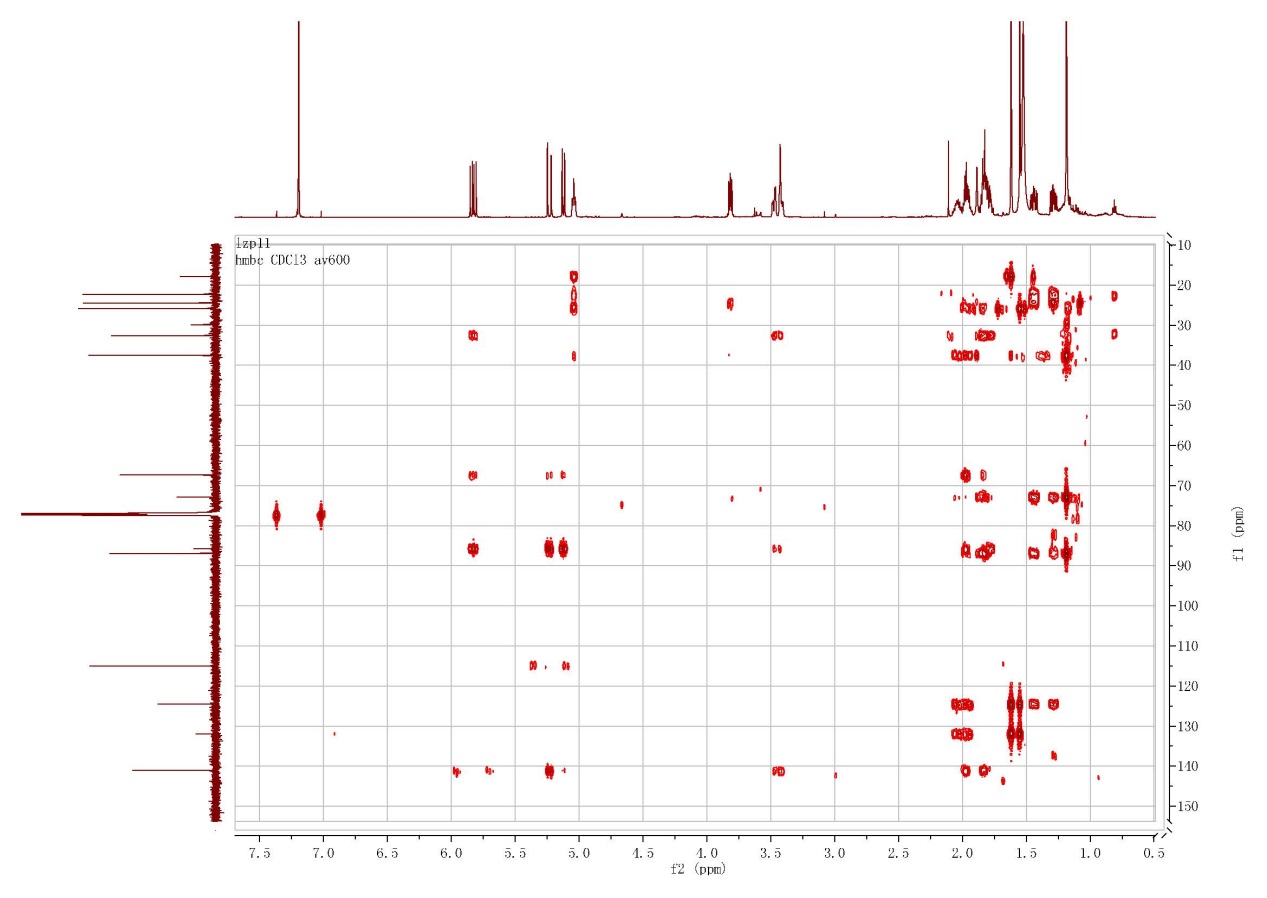

**Figure 21S.** ^1^H-^1^H COSY spectrum of compound **3** (600MHz, CDCl_3_).


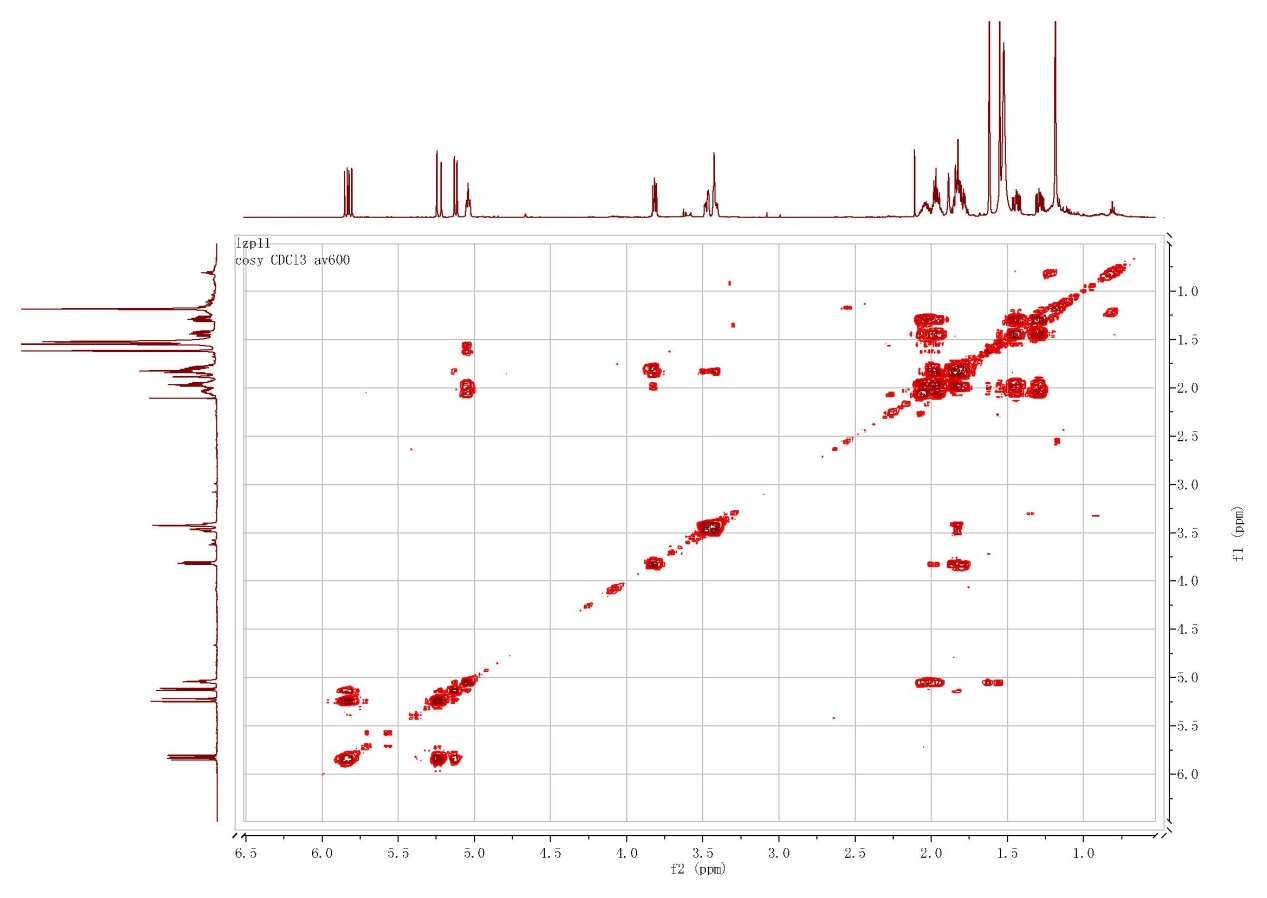

**Figure 22S.** ROESY spectrum of compound **3** (600MHz, CDCl_3_).


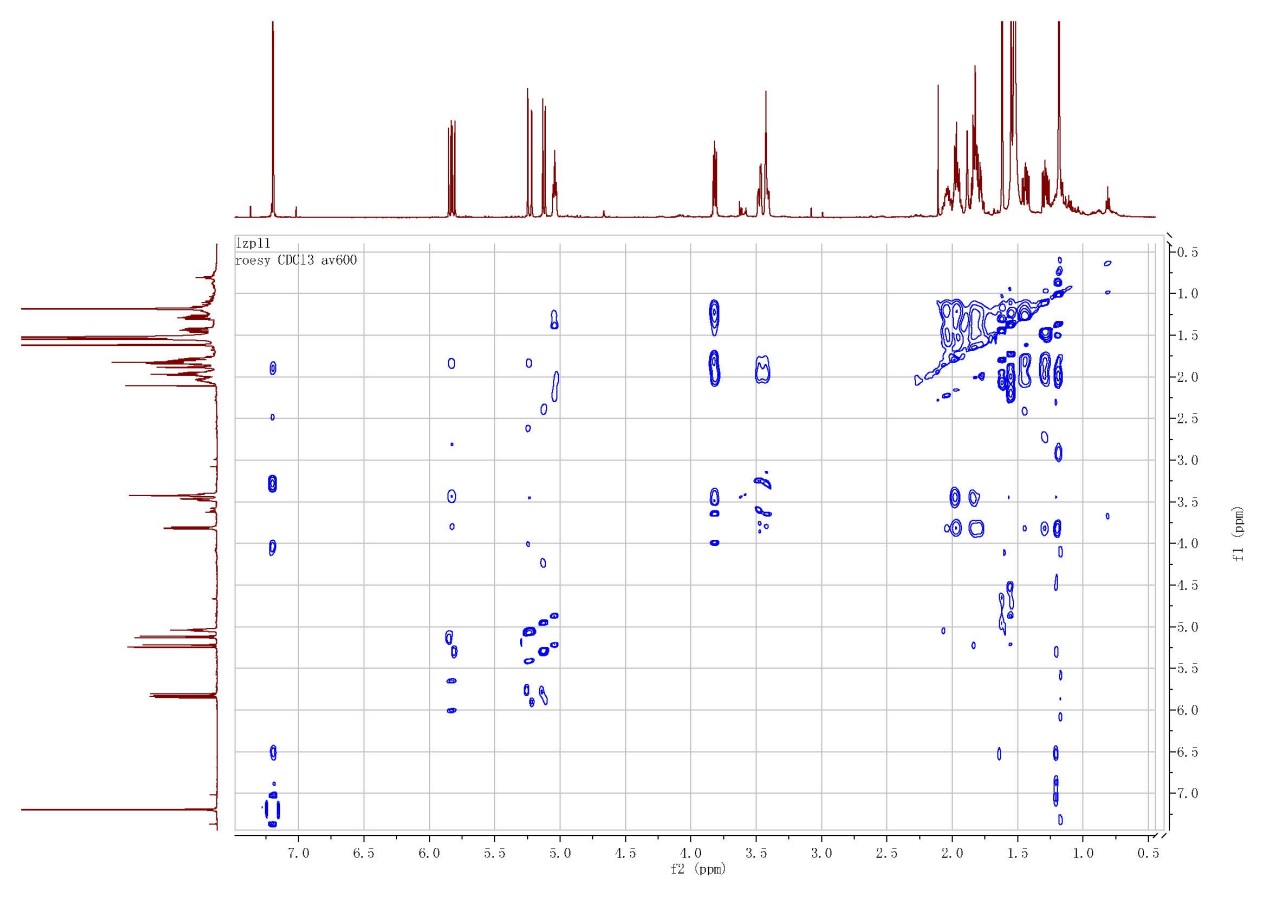

**Figure 23S.** HREIMS spectrum of compound **3**.


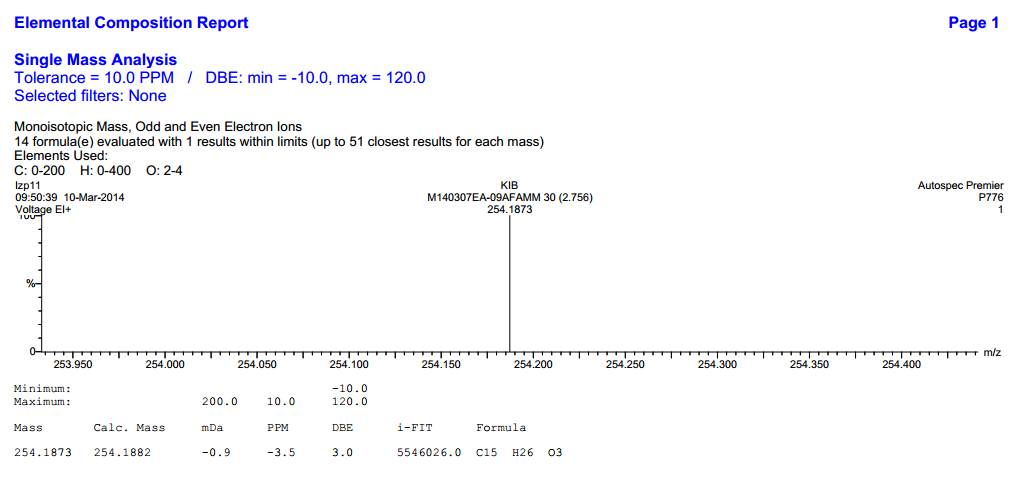


**Figure 24S.** ^1^HNMR spectrum of compound **4** (600MHz, CDCl_3_).


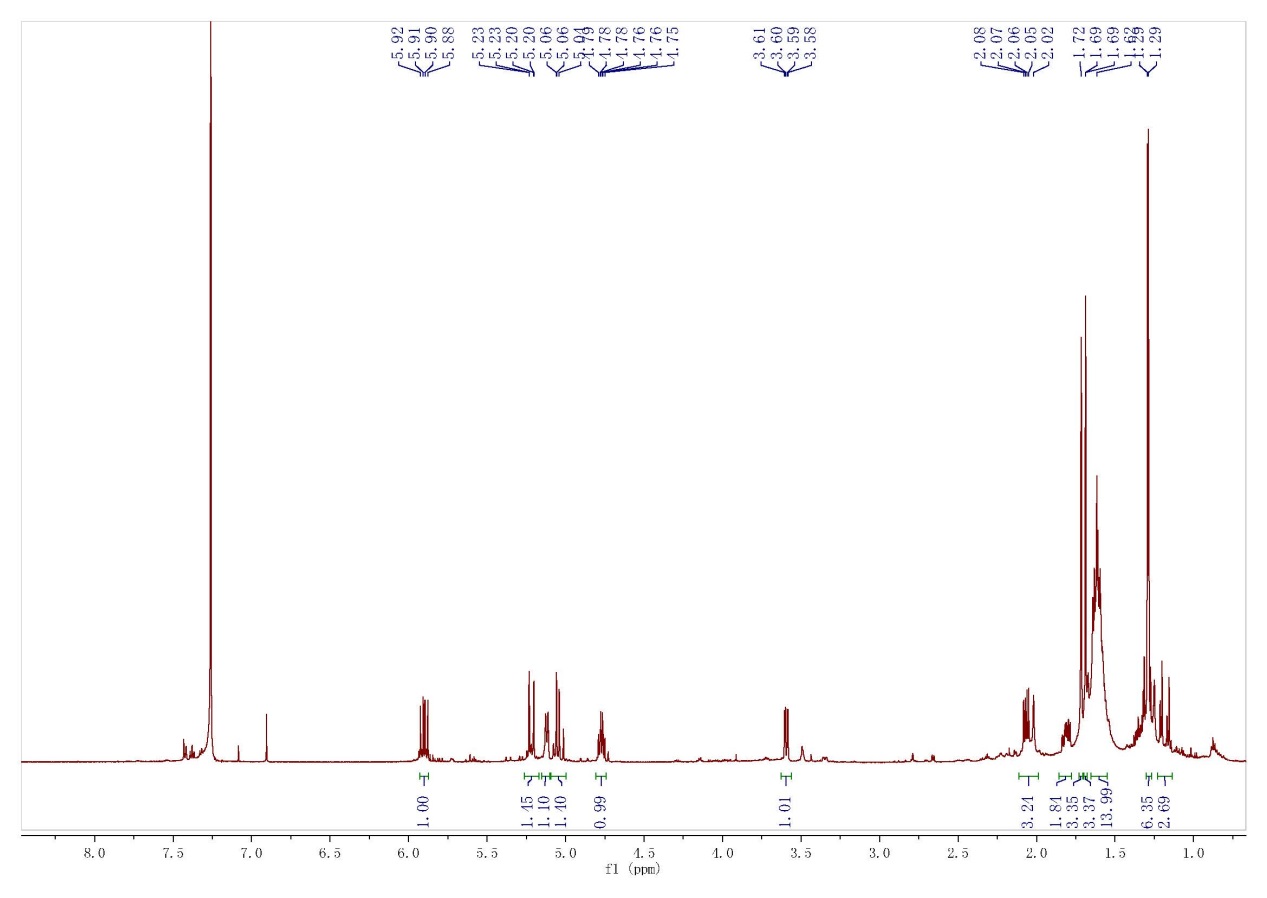

**Figure 25S.** ^13^CNMR spectrum of compound **4** (150MHz, CDCl_3_).


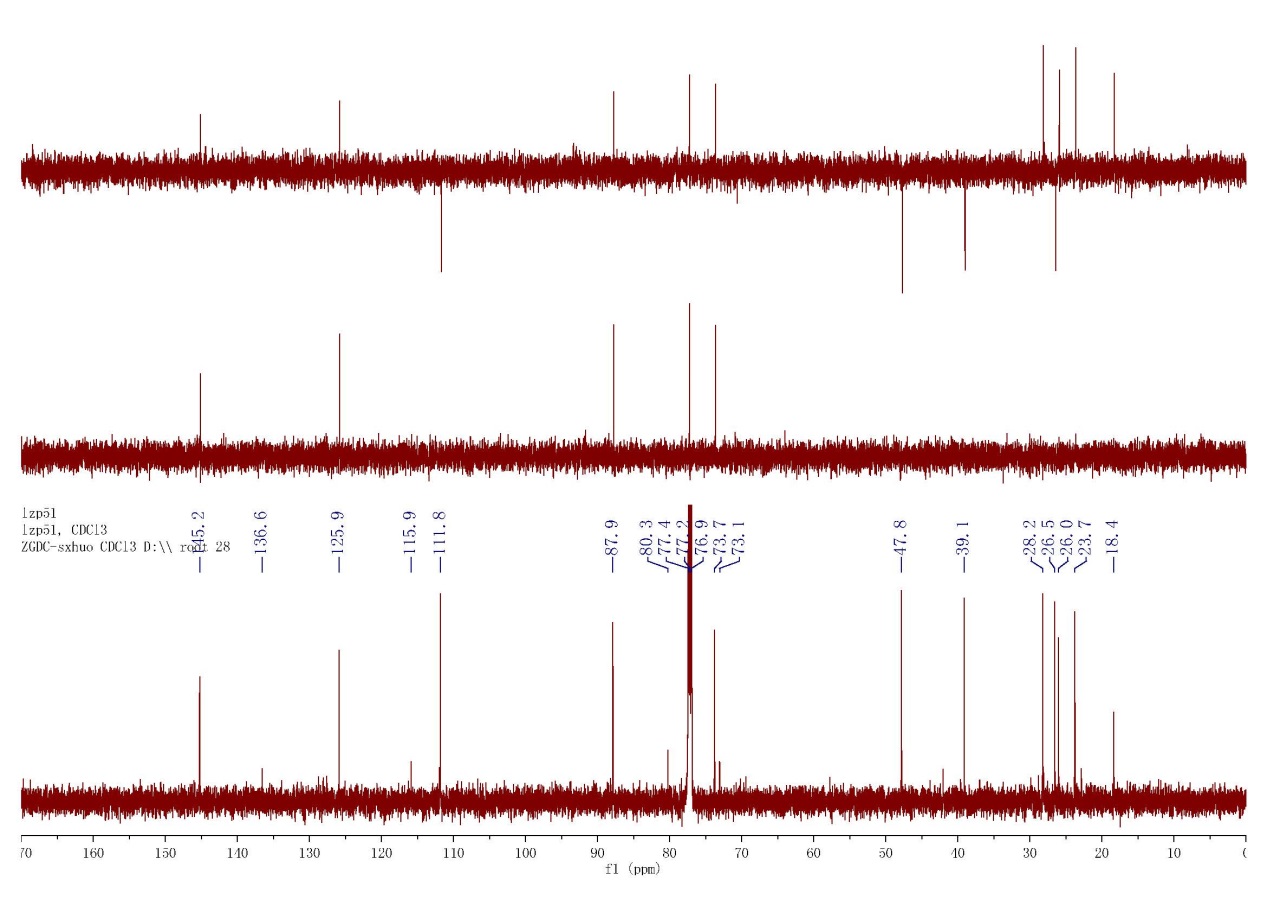

**Figure 26S.** HSQC spectrum of compound**4** (600MHz, CDCl_3_).


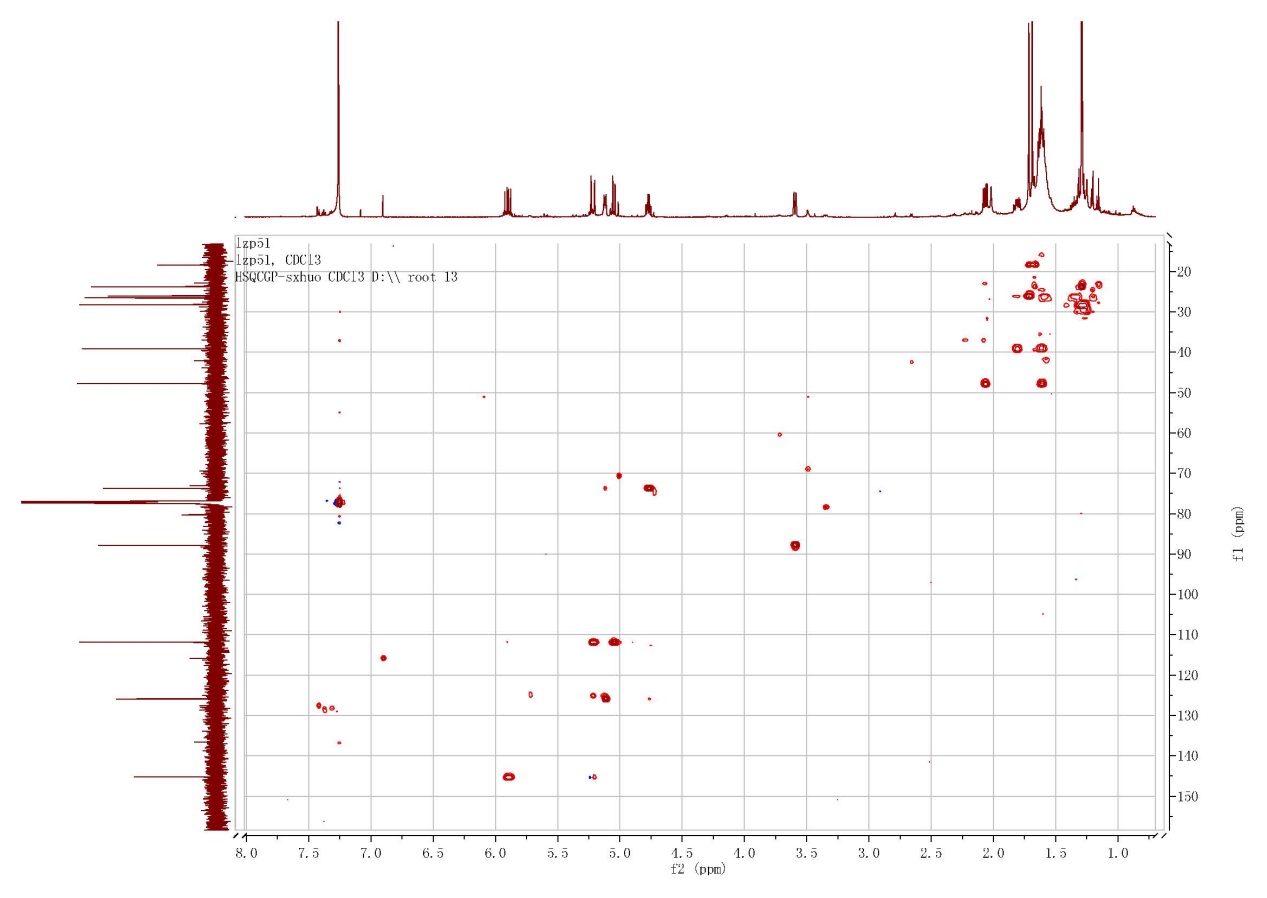

**Figure 27S.** HMBC spectrum of compound**4** (600MHz, CDCl_3_).


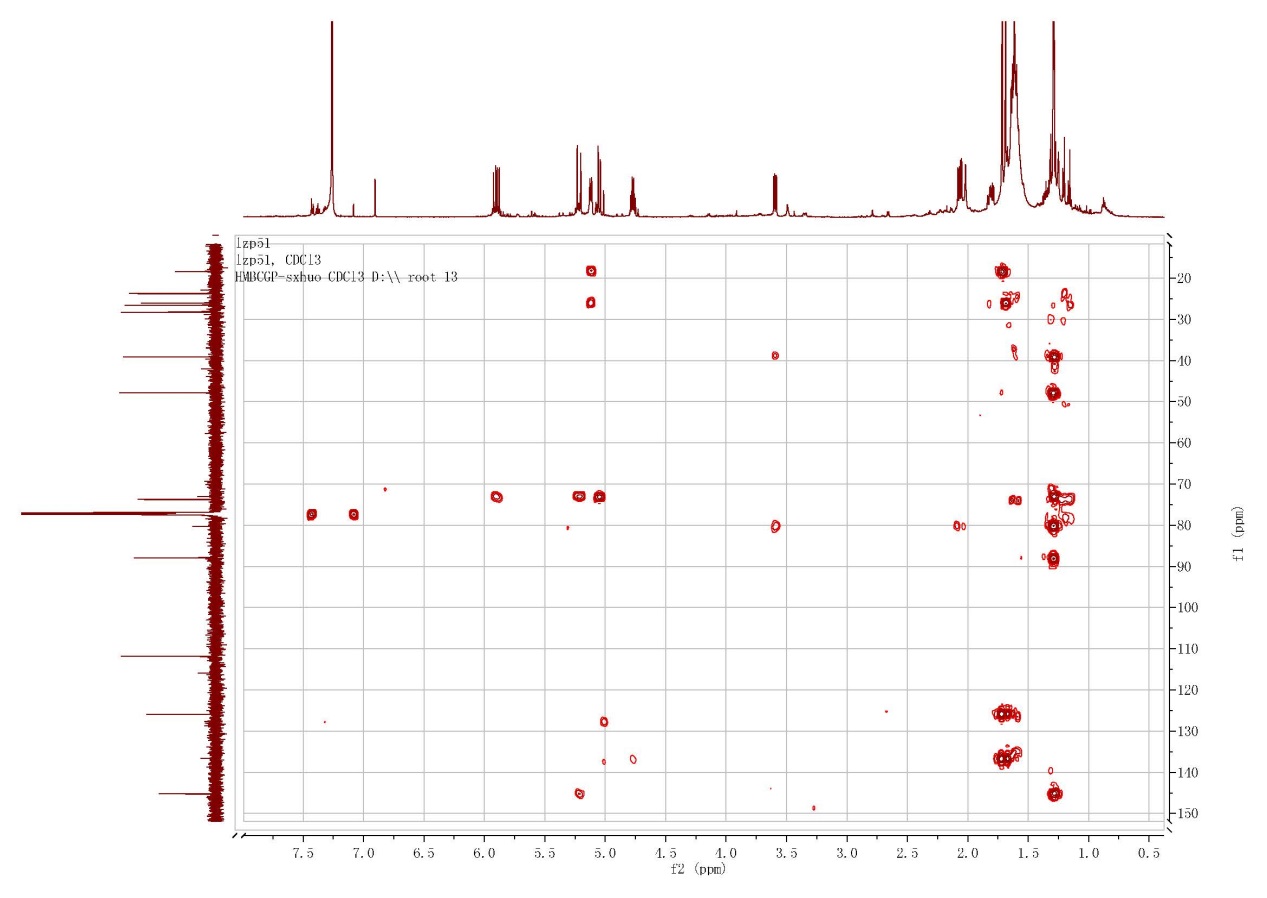

**Figure 28S.** ^1^H-^1^H COSY spectrum of compound**4** (600MHz, CDCl_3_).


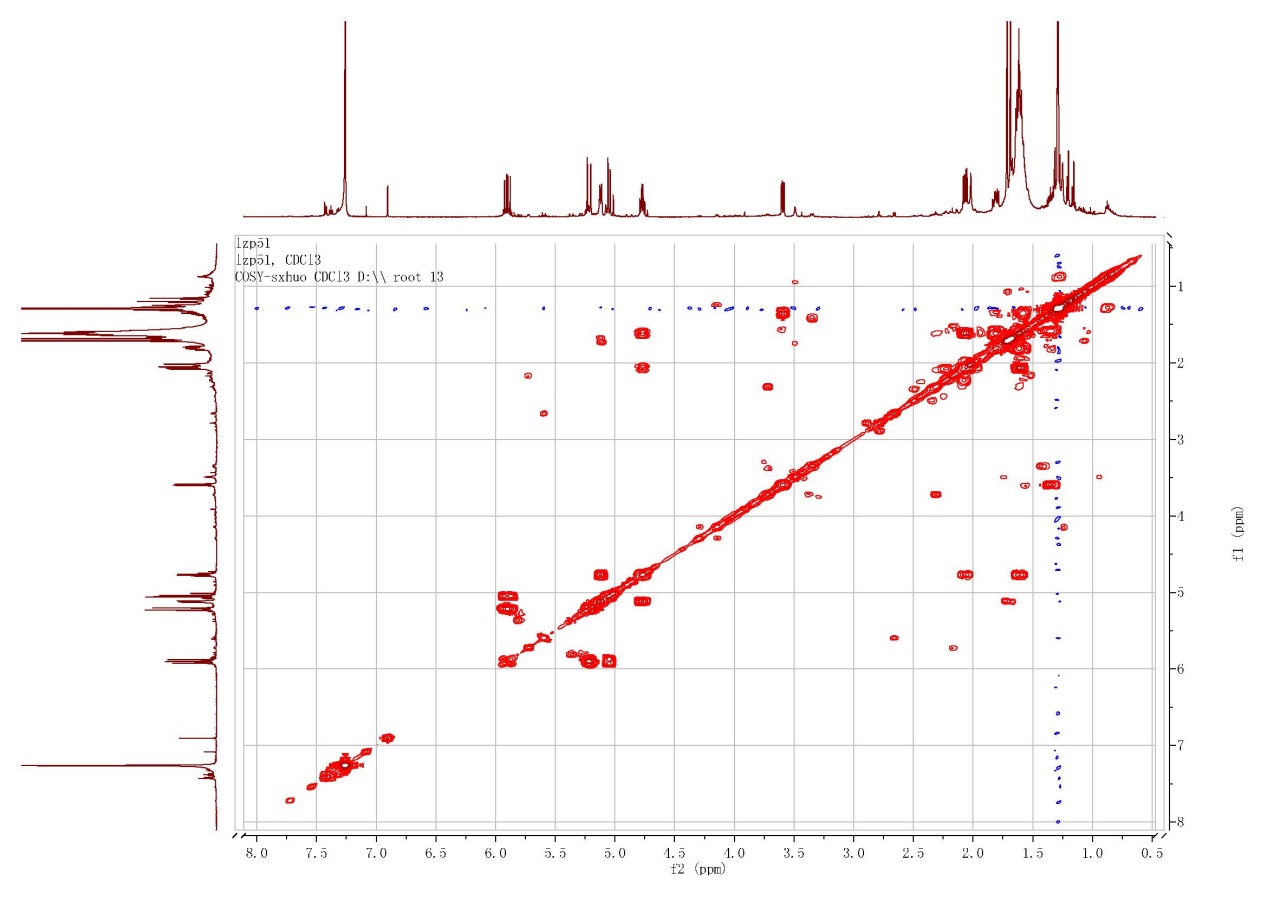

**Figure 29S.** ROESY spectrum of compound**4** (600MHz, CDCl_3_).


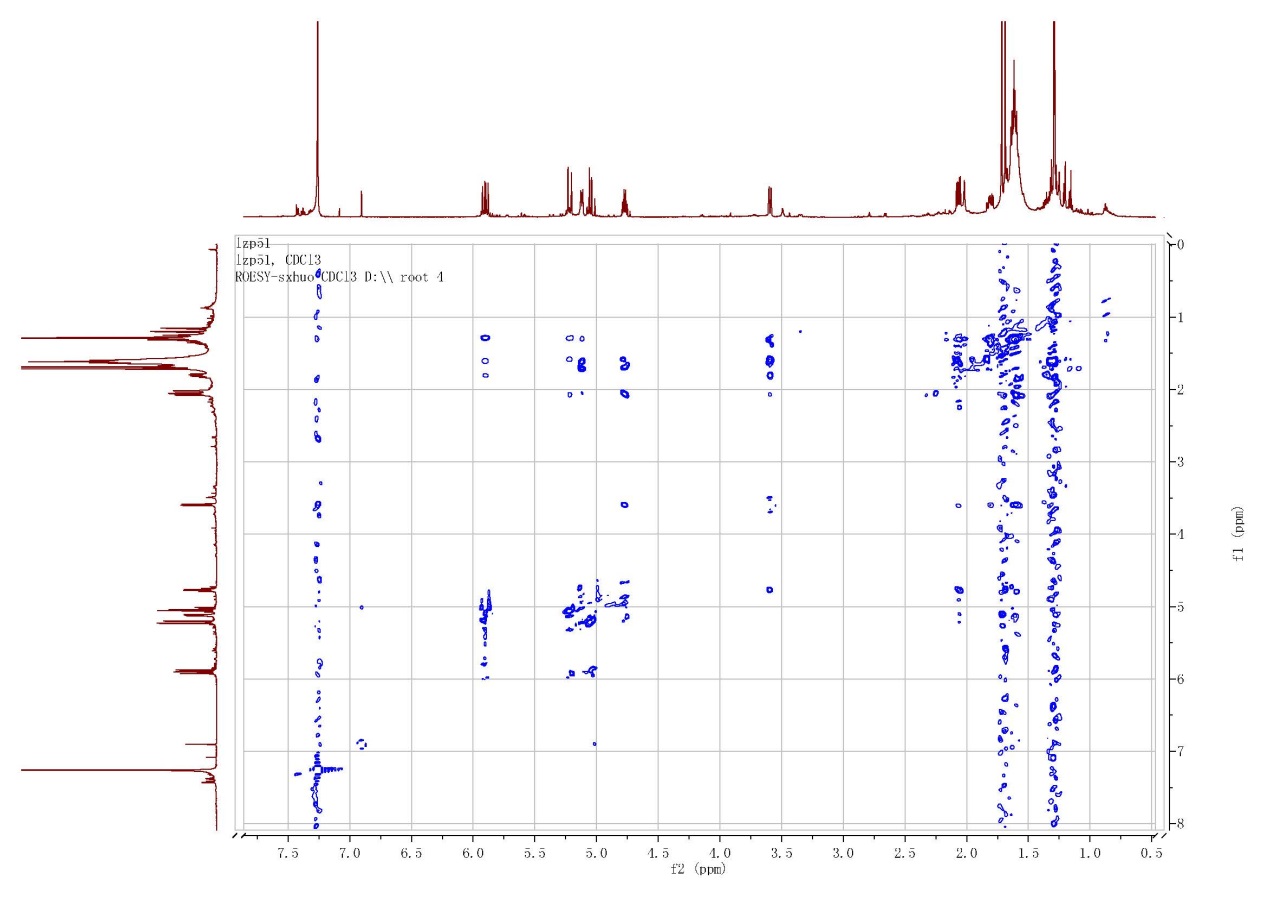

**Figure 30S.** HRESIMS spectrum of compound **4**.


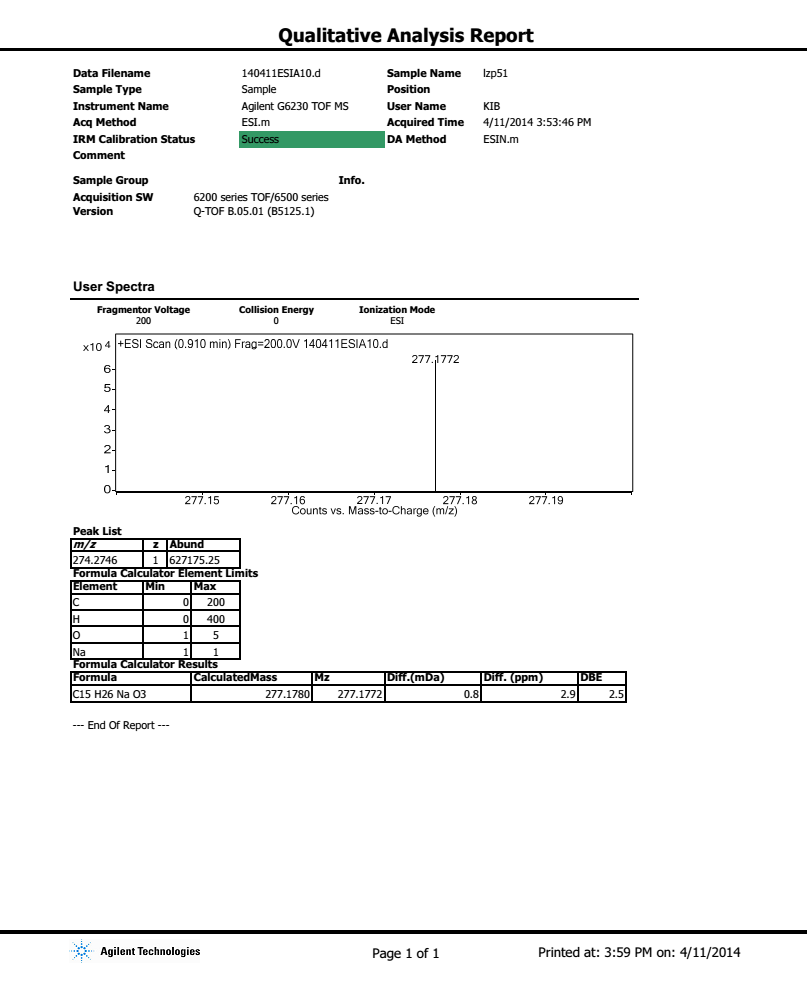


1. *Corresponding author. Kunming Institute of Botany, Chinese Academy of Sciences, Kunming 650201, P. R. China Tel.:+86 871 5216327; fax: +86 871 5150227.

   E-mail addresses:jkliu@mail.kib.ac.cn (J.K. Liu). [↑](#footnote-ref-2)
